# Systematic analysis of *PINK1* variants of unknown significance shows intact mitophagy function for most variants

**DOI:** 10.1038/s41531-021-00258-8

**Published:** 2021-12-10

**Authors:** Kai Yu Ma, Michiel R. Fokkens, Teus van Laar, Dineke S. Verbeek

**Affiliations:** 1 Department of Genetics, University of Groningen, University Medical Center Groningen, Groningen, The Netherlands; 2 Department of Neurology, University of Groningen, University Medical Center Groningen, Groningen, The Netherlands

**Keywords:** Parkinson's disease, Mutation, Genetic testing

## Abstract

Pathogenic variants in *PINK1* cause early-onset Parkinson’s disease. Although many *PINK1* variants have been reported, the clinical significance is uncertain for the majority of them. To gain insights into the consequences of *PINK1* missense variants in a systematic manner, we selected 50 *PINK1* missense variants from patient- and population-wide databases and systematically classified them using Sherloc, a comprehensive framework for variant interpretation based on ACMG-AMP guidelines. We then performed functional experiments, including mitophagy and Parkin recruitment assays, to assess the downstream consequences of *PINK1* variants. Analysis of *PINK1* missense variants based on Sherloc showed that the patient databases over-annotate variants as *likely pathogenic*. Furthermore, our study shows that *pathogenic PINK1* variants are most often linked to a loss-of-function for mitophagy and Parkin recruitment, while this is not observed for variants of unknown significance. In addition to the Sherloc framework, the added layer of evidence of our functional tests suggests a reclassification of 9/50 missense variants. In conclusion, we suggest the assessment of multiple layers of evidence, including functional data on top of available clinical and population-based data, to support the clinical classification of a variant and show that the presence of a missense variant in *PINK1* in a Parkinson’s disease case does not automatically imply pathogenicity.

## Introduction

Parkinson’s disease (PD) is the second most common neurodegenerative disorder worldwide, characterized by neuronal loss, with a focus on dopaminergic neurons in the substantia nigra^1^. Most PD patients have an idiopathic form of the disease that has an age at onset above 60 years. However, a small minority of patients (±4%) develop early-onset PD (EOPD) that begins before 45 years of age. For the majority of EOPD patients, the disease is inherited in an autosomal recessive manner via variants in the *PRKN*, *PINK1*, or *PARK7* genes that lead to a loss-of-function^2,3^. These genes are therefore often the first inspected using gene panels in genetic diagnostics of EOPD cases, with variants occasionally identified. With the advent of next-generation sequencing methods, an increasing number of sequence variants in these genes have been collected^4–7^. For instance, more than 300 variants of the *PINK1* gene have been identified. However, the functional consequences and clinical significance of the majority of these variants are unknown, which poses major challenges in genetic counseling practices.

To overcome hurdles in variant interpretation, the American College of Medical Genetics and Genomics and the Association for Molecular Pathology (ACMG-AMP)^8,9^ provide guidelines to systematically categorize pathogenicity using standard terminology. In addition, common frameworks such as Sherloc have been established on top of the guidelines set by the ACMG-AMP to objectively assess pathogenicity using specific scoring systems that minimize bias^10^. Variants that have been proven to be (likely) disease-causing based on clinical, population-wide and functional evidence are annotated as *(likely) pathogenic*, while variants that are (likely) not disease-causing based on these sources of evidence are annotated as *(likely) benign*. However, most variants cannot be clearly assigned to these groups because the evidence for *(likely) pathogenic* and *(likely) benign* are both insufficient and are thus designated as having *uncertain significance*. As there are still only few functional studies on rare variants, current annotations often rely heavily on data such as the minor allele frequency (MAF) of a variant in population cohorts and the segregation of the variant with disease (when available). *In silico* computational predictions and modeling using the corresponding protein structure may provide added guidance for the interpretation of variants. To more accurately categorize pathogenicity, we need multiple layers of evidence, including functional testing in the form of appropriate experimental assays that can determine the biochemical or cellular consequence of the variant. Although these steps are all necessary to improve genetic diagnostics, pathogenicity of variants may remain uncertain even when there is a multitude of different data. Recently, a study showed that only a minority of the numerous reported *PRKN* variants could be classified as *pathogenic* using Sherloc in combination with cellular functional studies^11^.

**Fig. 1:**
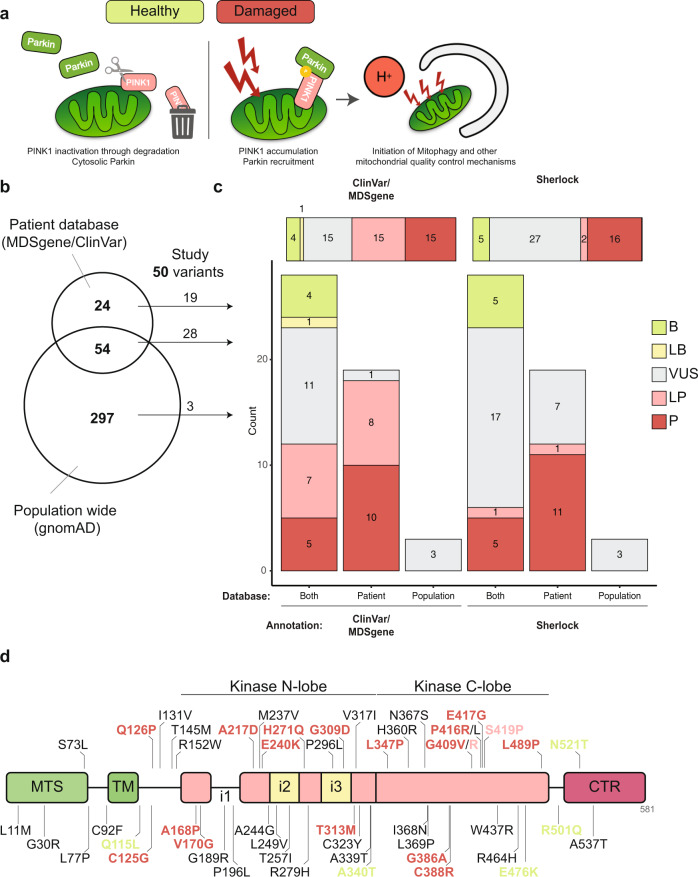
Most *PINK1* missense variants are classified as uncertain significance using Sherloc. **a** Schematic overview of the PINK1/Parkin pathway in healthy and damaged state. **b** Venn diagram showing the number of unique and overlapping *PINK1* missense variants reported in disease-related databases (ClinVar and MDSgene) and the population-wide database gnomAD. **c** ACMG-AMP annotations of the 50 missense variants investigated in this study, as determined by the disease-related databases (left) or using Sherloc (right). Horizontal bar graphs show all the studied variants. Vertical bar graphs are stratified by the origin of the reported missense variant in (**b**). **d** Schematic overview of the functional domains of PINK1 with the location of the 50 missense variants. Variants are color-coded following the legend of (**c**). B = benign; LB = likely benign; VUS = variant of uncertain significance; LP = likely pathogenic; P = pathogenic; MTS = mitochondrial targeting sequence; TM = transmembrane domain; CTR = C-terminal region.

*PINK1* is another example of a recessive PD gene with many reported genetic variants. *PINK1* encodes PTEN-induced putative kinase 1 (PINK1), a serine-threonine kinase that works in concert with the E3 ubiquitin ligase Parkin (encoded by *PRKN*) to maintain mitochondrial quality^12–15^. Through a membrane potential-dependent process, PINK1 is imported from the outer mitochondrial membrane (OMM) into the inner mitochondrial membrane, where it is constitutively degraded by mitochondrial proteases^16,17^. However, PINK1 import and cleavage is blocked upon mitochondrial depolarization caused by damage (e.g. oxidative stress), resulting in the accumulation of PINK1 on the OMM. At the OMM, PINK1 activates Parkin through phosphorylation of Parkin and ubiquitin, leading to stable recruitment and activation of Parkin onto the mitochondrial surface^17–19^. By ubiquitinating different OMM substrates, Parkin then orchestrates different mitochondrial quality control pathways, from the degradation of individual proteins to the complete removal of damaged mitochondria via mitophagy (Fig. 1a)^13–15^.

In the present study, we utilized the Sherloc framework to systematically annotate 50 missense variants in *PINK1* and followed this up with functional in vitro assays to gain insights into the consequence of these variants, with the overall aim being to provide better guidance for the interpretation of rare and/or novel missense variants.

## Results

### Compilation of disease-associated and population-based missense variants in *PINK1*

To compile an overview of the *PINK1* missense variants reported in disease-associated databases and population-based databases, we searched the Movement Disorder Society Genetic mutation database (MDSgene)^5^ and ClinVar^6^ databases for missense variants in *PINK1* and identified 78 variants reported in EOPD or PD patients, of which 54 were also reported in the population-based Genome Aggregation Database (gnomAD)^7^ (Fig. 1b). For practical reasons, we selected 46 disease-associated variants (this included all *pathogenic* variants), of which 19 were not reported in gnomAD, and four rare population-based missense variants (<8 reported alleles) to obtain a list of missense variants that covers the whole coding region of *PINK1* (Fig. 1b). Although the clinical significance of each of these 46 missense variants was assigned by the patient databases, the pathogenic evaluation and the presence of bias in the interpretation remained unclear. The reported clinical significance of these 46 missense variants showed enrichment for *likely pathogenic* (15/46) and *pathogenic* (15/46) and an underrepresentation of *likely benign* (1/46) and *benign* (4/46) variants, while 11/46 variants were of *uncertain significance*. The rare population-based variants were classified with *uncertain significance* (Fig. 1c).

### Many disease-associated missense variants in *PINK1* are of *uncertain significance*

In order to better classify these 50 missense variants, we used the most recent best practice guidelines for variant classification recommended by the ACMG-AMP^9^. Pathogenicity was determined based on several criteria with varying strengths of evidence from which the annotation is derived^9^. However, since several criteria could not be checked with certainty or were not applicable for the missense variants in this study (Supplementary Fig. 1), most variants remained of *uncertain significance* (Supplementary Table 1). To overcome this, we utilized the semiquantitative Sherloc framework, a comprehensive refinement of the ACMG-AMP criteria^10^, to re-analyze the clinical significance of the missense variants. Benign and pathogenic points were allocated to each variant based on four layers of evidence (Supplementary Fig. 2): (1) the rarity of the variant based on MAF and homozygosity status derived from population-wide databases gnomAD/dbSNP, (2) whether the variant was found in patients, derived from disease-associated databases ClinVar/MDSgene, (3) segregation of the variant based on detailed family-reports from literature, and (4) evidence from experimental studies collected from the literature (Supplementary Table 2). Supplementary Table 1 provides a detailed overview of the points assigned per missense variant, including the summed benign and pathogenic points and the clinical significance.

Using the Sherloc framework, ten *likely pathogenic* variants and only two *pathogenic* variants were reclassified to *uncertain significance*, but no *benign* variants were. Furthermore, three *likely pathogenic* variants became *pathogenic*, and the one *likely benign* variant became *benign*. No variants of *uncertain significance* were reclassified (Fig. 1c). Not unexpectedly, missense variants reported to be unique in patients were mostly classified as *pathogenic* (11/19; Fig. 1c), and variants present in both patient and population databases were mostly classified as *uncertain significance* (17/28) or *benign* (5/28), with fewer being classified as *likely pathogenic* (1/28) or *pathogenic* (5/28). Thus, based on our Sherloc reclassification, most missense variants in *PINK1* that are reported in disease-associated databases are of *uncertain significance*.

### Generation of a mitophagy reporter to study the consequences of PINK1 carrying missense variants

We then investigated if variants classified as *pathogenic* are more often located or specifically located in one of the several domains of PINK1, including the N-terminal mitochondrial targeting signal, transmembrane domain, and the kinase domain with three insertions^20^. Interestingly, we observed that most of the *pathogenic* variants (14/16) are located in the kinase domain (Fig. 1d), suggesting a critical role for this domain. Indeed, previous studies have shown that many *pathogenic* variants (as classified in these earlier studies) abrogate the kinase activity of PINK1, leading to a failure in Parkin recruitment to mitochondria upon mitochondrial damage (Supplementary Table 2)^18,21,22^. Consequently, PINK1 and Parkin are unable to activate mitophagy-mediating proteins at the OMM, leading to failure in mitophagy induction^13^.

We then hypothesized that we could use mitophagy induction as a read-out of PINK1 function/activity and that the results of this experimental study could add to the fourth layer of evidence in the Sherloc framework, thereby supplementing existing evidence for the 50 missense variants. This would allow us to further improve the classification of these variants. To do so, we generated a mitophagy reporter in Human cervix epithelioid carcinoma (HeLa) cells in which we genetically depleted *PINK1* using Clustered Regularly Interspaced Short Palindromic Repeats (CRISPR)-Cas9, after which we stably expressed a reporter construct with the fluorophore mt-mKeima and FLAG-Parkin (Supplementary Fig. 3). mt-mKeima allows for sensitive quantification of mitochondria in the cytosol and lysosome^23^, whereas FLAG-Parkin is necessary to study PINK-Parkin-induced mitophagy as HeLa cells do not express endogenous Parkin (Supplementary Fig. 3C).

### Most (*likely*) *pathogenic* PINK1 variants affect mitophagy induction

**Fig. 2:**
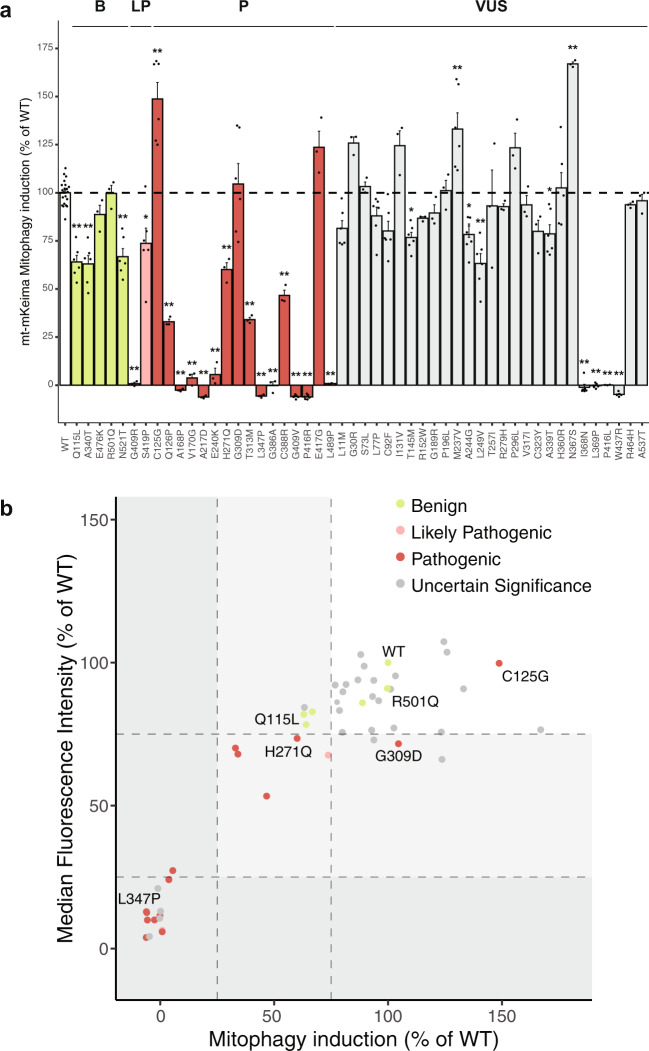
Mitophagy induction is affected by most (*likely*) *pathogenic* PINK1 variants. **a** Assessment of mitophagy induction upon CCCP treatment in HeLa PINK1 KO cells stably expressing mt-mKeima-FLAG-Parkin transfected with PINK1 WT or missense variants. All data shown are normalized against PINK1 WT control. Each dot represents a separate experiment. Bars are color-coded by the annotation as determined by Sherloc. Data analyzed using one-way ANOVA. *p < 0.05, ** *p* < 0.005. **b** Relation between mitophagy induction and median fluorescence intensity of acidic mt-mKeima for PINK1 WT or missense variants. The *X*-axis is mean data from (**a**). *Y*-axis is mean data from Supplementary Fig. 4B. Three groups of variants based on the amount of mitophagy function are highlighted in the areas in white (group 1), light gray (group 2), and dark gray (group 3). Each dot represents a variant and is color-coded by the Sherloc annotation.

To study PINK1-Parkin mitophagy, we treated the reporter cells transiently expressing wild type (WT) PINK1-HA or variant-PINK1-HA with protonophore carbonyl cyanide 3-chlorophenylhydrazone (CCCP), which induces mitochondrial stress by dissipating the mitochondrial membrane potential that leads to the stabilization of PINK1 and Parkin recruitment to mitochondria^17^. After 24 h of 10 µM CCCP treatment, mitophagy induction was determined using FACS. CCCP treatment in WT-PINK1-HA-expressing reporter cells led to an increase in cells with mitochondria in acidic compartments (i.e., lysosomes), indicative of mitophagy induction (Fig. 2a; Supplementary Fig. 4A.i/ii). When we transiently expressed the different variant-PINK1-HA proteins, we observed that the majority of missense variants in *PINK1* resulted in a wide range of mitophagy induction (Fig. 2a) that was not caused by differences in their protein levels, as assessed by western blotting (Supplementary Fig. 5A, B), except for variant p.Val317Ile that showed significantly increased PINK1 protein levels, which might possibly mask a reduced mitophagy induction due to loss of PINK1 kinase activity.

We found that the PINK1 proteins carrying missense variants classified as *benign* all induced mitophagy upon CCCP treatment, although some did show a slightly decreased function (p.Gln115Leu, p.Ala340Thr, p.Asn521Thr) compared to WT PINK1 (Fig. 2a). In contrast, most missense variants in *PINK1* annotated as *(likely) pathogenic* either caused a significant decrease in mitophagy induction (p.Ser419Pro, p.Gln126Pro, p.His271Gln, p.Thr313Met, and p.Cys388Arg) compared to WT PINK1 or completely abolished mitophagy induction (p.Gly409Arg, p.Ala168Pro, p.Val170Gly, p.Ala217Asp, p.Glu240Lys, p.Leu347Pro, p.Gly386Ala, p.Gly409Val, and p.Leu489Pro) upon CCCP treatment (Fig. 2a). Interestingly, PINK1 carrying the *pathogenic* missense variants p.Gly309Asp and p.Glu417Gly was able to induce mitophagy in a manner similar to WT PINK1, while PINK1 carrying the *pathogenic* missense variant p.Cys125Gly increased mitophagy induction compared to WT PINK1 upon CCCP treatment. This suggests that these missense variants are either not *pathogenic* or exert a different pathogenic mechanism that does not involve mitophagy. Four missense variants of *uncertain significance* in PINK1 (p.Thr145Met, p.Ala244Gly, p.Leu249Val, and p.Ala339Thr) exhibited a significant decrease compared to WT PINK1 upon CCCP treatment. Notably, four missense variants (p.Ile368Asn, p.Leu369Pro, p.Pro416Leu, and p.Trp437Arg) that were classified as *uncertain significance* completely abolished mitophagy induction. Based on this data, we conclude that these latter four variants affect PINK1 function that may lead to their reclassification as *likely pathogenic*. Overall, most missense variants of *uncertain significance* caused no significant alteration in mitophagy induction, and thus, using the mitophagy reporter, we found no additional evidence to reclassify these variants.

In addition, for some PINK1 variants, we observed that the mt-mKeima fluorescence signal was reduced even though the induction of mitophagy was unaltered compared to WT PINK1 treated with CCCP (e.g., Gly309Asp, Supplementary Fig. 4A.vii). Therefore, we determined the median fluorescence intensity (MFI) of mt-mKeima of cells expressing the variants of PINK1 after CCCP treatment. For most cells expressing variant PINK1, the MFI increased upon CCCP treatment in accordance with an increase in mitophagy induction (Fig. 2b, Supplementary Fig. 4B). However, a marked decrease in MFI was observed in cells expressing PINK1 carrying several variants (including p.Gly309Asp, p.Glu417Gly, p.Arg279His, p.Pro296Leu, p.Cys323Tyr, p.His360Arg, p.Asn367Ser, and p.Arg464His) that showed normal or increased mitophagy induction. This likely suggests that these missense variants do affect PINK1 function and lead to a decrease in mitophagy function. When using both mitophagy induction and MFI, three groups of missense variants were distinguished: (1) missense variants with normal or increased mitophagy function (Fig. 2b, white area), (2) missense variants that cause decreased (25–75%) mitophagy function (Fig. 2b, light gray area) and (3) missense variants that completely abolish mitophagy function (Fig. 2b, dark gray area). Importantly, *(likely) pathogenic* missense variants fell mostly in groups 2 and 3, further confirming that mitophagy deficits underlie PINK1 dysfunction.

### Many PINK1 variants affect Parkin translocation

**Fig. 3:**
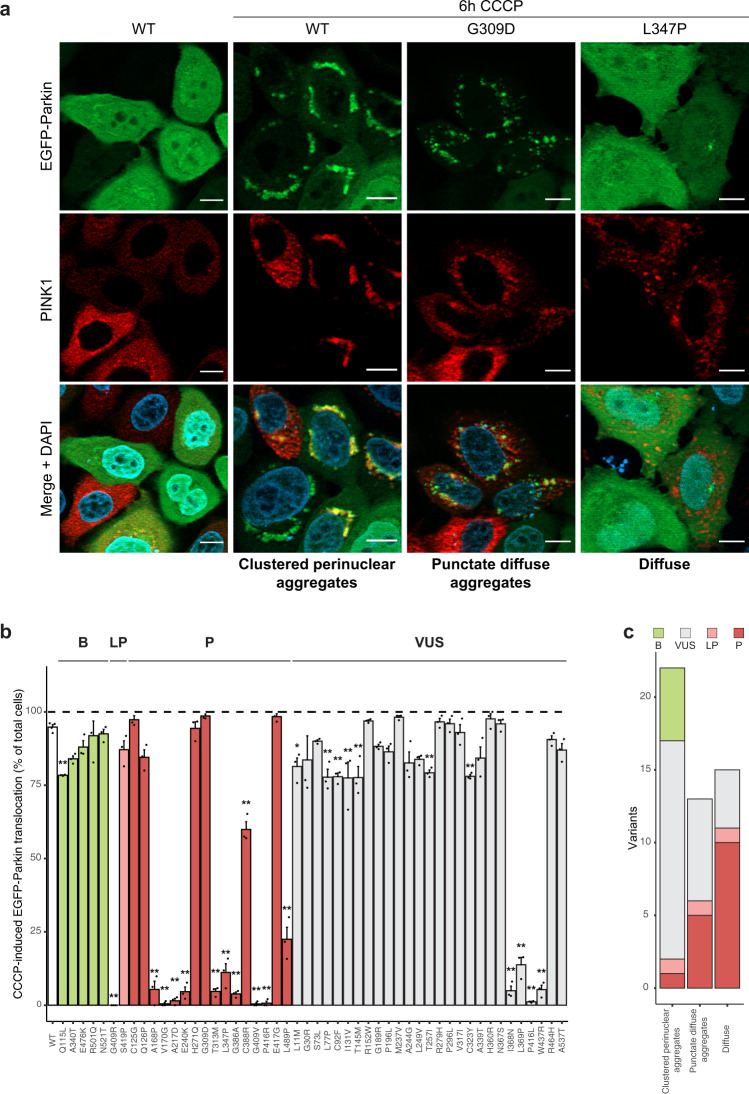
PINK1 variants with disrupted mitophagy affect Parkin translocation. **a** Representative fluorescence images of HeLa PINK1 KO cells stably expressing EGFP-Parkin transfected with PINK1-HA WT and PINK1-HA variants p.Gly309Asp and p.Leu347Pro and before or after treatment with CCCP showing examples of three types of Parkin localization: clustered perinuclear aggregates, punctate diffuse aggregates, and diffuse cytosolic localization. Upper panels show EGFP-Parkin, middle panels show PINK1, and lower panels show an overlay including DAPI to stain nuclei. Scale bar, 10 μm. **b** Quantification of Parkin translocation by PINK1 WT or PINK1 missense variants upon CCCP treatment. Mean values are shown from three independent experiments. Bars are color-coded by the annotation as determined by Sherloc. Data analyzed using one-way ANOVA. *p < 0.05, **p < 0.005. **c** Quantification of variants for each of the types of Parkin localization, color-coded by the Sherloc annotation.

To further confirm that variant PINK1 mitophagy function was indeed affected via PINK1-Parkin‒dependent mechanisms^15,17,18^, we also investigated the mitochondrial translocation of Parkin. To this end, we transiently transfected WT-HA-tagged PINK1 (PINK1-HA) or variant-PINK1-HA in stably expressed EGFP-Parkin/PINK1 knock-out (KO) HeLa cells treated with 10 µM CCCP for 6 hours following immunofluorescence. CCCP treatment caused translocation of cytosolic EGFP-Parkin into “clustered perinuclear aggregates” (representing mitochondria^17^) that colocalized with WT PINK1 (Fig. 3a/b), as was also observed for all PINK1 proteins carrying *benign* variants. In contrast, cells expressing PINK1 variants designated as *(likely) pathogenic* had aberrant EGFP-Parkin localization that was mainly in the form of “punctate diffuse aggregates”, such as p.Gly309Asp (Fig. 3a), or remained “diffuse”, such as p.Leu347Pro (Fig. 3a). The latter correlated completely with abolished mitophagy induction (Fig. 2a). “Diffuse” EGFP-Parkin was also observed for four of the missense variants of *uncertain significance* that also showed abolished mitophagy induction (Fig. 2a). Interestingly, cells expressing PINK1 proteins carrying missense variants (mainly of *uncertain significance*) that showed “punctate diffuse aggregates” of EGFP-Parkin (p.Gln126Pro, p.Ile131Val, p.Met237Val, p.Leu249Val, p.His271Gln, p.Pro296Leu, p.Gly309Asp, p.His360Arg, p.Asn367Ser, p.Cys388Arg, p.Glu417Gly, p.Ser419Pro, and p.Arg464His, Supplementary Fig. 6) did induce mitophagy upon CCCP treatment, albeit some at decreased levels (Fig. 2a). Since mitochondrial clustering occurs as a result of the formation of poly-ubiquitin chains by Parkin^15^, we hypothesize that these missense variants (despite mitophagy induction) do impair PINK1 function, thereby affecting Parkin recruitment to mitochondria upon CCCP treatment, and thus may exert *likely pathogenic* effects. Overall, missense variants that caused a decrease in mitophagy function also led to impairments in EGFP-Parkin translocation, while variants with normal mitophagy function caused complete translocation of cytosolic EGFP-Parkin (Fig. 3b, c).

### Functional assays assist in the determination of pathogenicity but do not guarantee classification

**Fig. 4:**
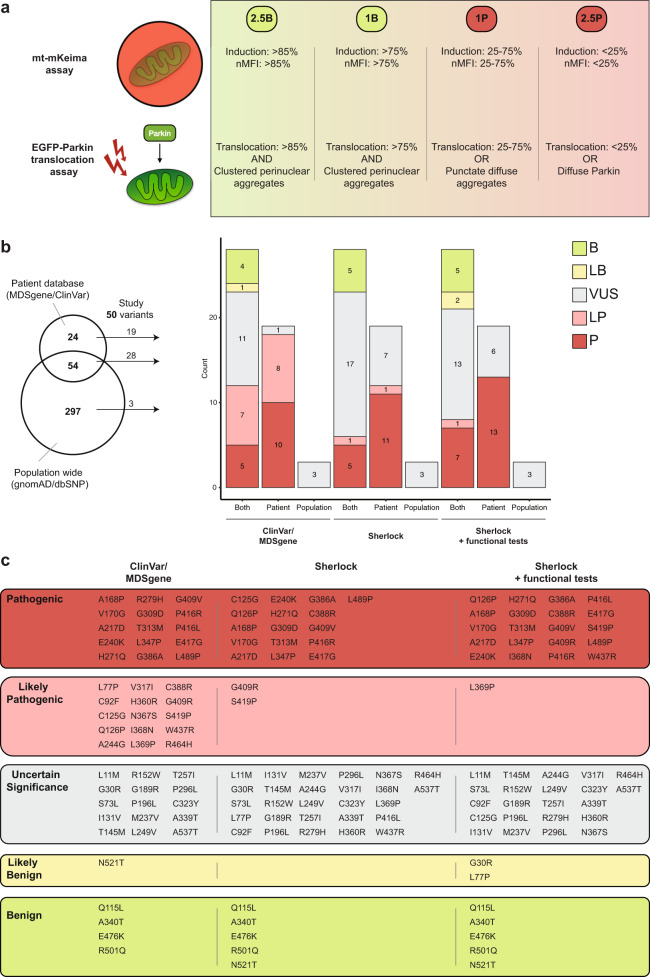
Updating Sherloc-based classification with functional assay results in the reclassification of nine *PINK1* variants. **a** Scoring system based on the functional assays in this study. Points were assigned as shown, based on both the mitophagy assay (%induction and MFI) and the Parkin translocation assay (% translocation and type of Parkin translocation). **b** Updated ACMG-AMP annotations of the 50 missense variants as shown in Fig. 1c, including the updated Sherloc classification (right). Horizontal bar graphs show all the studied variants. Vertical bar graphs are stratified by the origin of the reported missense variant in the Venn diagram shown on the left. **c** Overview of all *PINK1* missense variants assessed in this study and their annotation based on the patient databases, Sherloc and Sherloc after functional testing.

Since the PINK1 variants had varying functional consequences, we constructed a scoring system based on the observed PINK1 deficits (Fig. 4a). Specifically, points were assigned in a similar manner to the Sherloc framework (Supplementary Fig. 1D): 2.5 *benign* points = PINK1-variant similar to WT PINK1, 2.5 *pathogenic* points = PINK1-variant disables mitophagy function and Parkin translocation, one *pathogenic* point = PINK1-variant affects mitophagy function or Parkin translocation, or one *benign* point = PINK1-variant mildly reduced mitophagy function or Parkin localization compared to WT PINK1. Next, we complemented the original Sherloc scores with our functional assay scores. This led to a revision of the final classification for nine variants (9/50 = 18%) (Fig. 4b): six variants of *uncertain significance* were reclassified to *pathogenic* (p.Ile368Asn, p.Gly409Arg, p.Pro416Leu, p.Ser419Pro, and p.Trp437Arg) or *likely pathogenic* (p.Leu369Pro), two variants of *uncertain significance* (p.Gly30Arg and p.Leu77Pro) were reclassified to *likely benign*, and one *pathogenic* variant (p.Cys125Gly) was reclassified to *uncertain significance*. The classification of the benign variants and the four rare population-based variants did not change. In total, based on our data, 20 of the 46 clinically relevant variants are classified as *pathogenic*, one as *likely pathogenic*, two as *likely benign*, and five as *benign* (Table 1, Fig. 4c, Supplementary Fig. 7).

**Table 1:** Final *PINK1* variant reclassification using Sherloc and functional assays.

*PINK1* -Variant	Database	Clinical assignment			Final Sherloc score	
		ClinVar/MDSgene	Sherloc	Sherloc + assays	P	B
p.Leu11Met	ClinVar	Uncertain significance	Uncertain significance	Uncertain significance	1	1
p.Gly30Arg	ClinVar, gnomAD	Uncertain significance	Uncertain significance	Likely Benign	–	4
p.Ser73Leu	ClinVar, MDSgene, gnomAD	Uncertain significance	Uncertain significance	Likely Benign	–	3.5
p.Leu77Pro	MDSgene	Likely Pathogenic	Uncertain significance	Uncertain significance	3	1
p.Cys92Phe	MDSgene	Likely Pathogenic	Uncertain significance	Uncertain significance	3.5	1
p.Gln115Leu	ClinVar, gnomAD	Benign	Benign	Benign		7
p.Cys125Gly	MDSgene	Likely Pathogenic	Pathogenic	Uncertain significance	2.5	–
p.Gln126Pro	MDSgene, gnomAD	Likely Pathogenic	Pathogenic	Pathogenic	9.5	–
p.Ile131Val	gnomAD	Uncertain significance	Uncertain significance	Uncertain significance	0.5	–
p.Thr145Met	ClinVar, gnomAD,	Uncertain significance	Uncertain significance	Uncertain significance	0.5	2.5
p.Arg152Trp	ClinVar, gnomAD	Uncertain significance	Uncertain significance	Uncertain significance	2	2.5
p.Ala168Pro	ClinVar, MDSgene, gnomAD	Pathogenic	Pathogenic	Pathogenic	6.5	–
p.Val170Gly	MDSgene	Pathogenic	Pathogenic	Pathogenic	5.5	–
p.Gly189Arg	ClinVar, gnomAD	Uncertain significance	Uncertain significance	Uncertain significance	–	1
p.Pro196Leu	ClinVar, gnomAD	Uncertain significance	Uncertain significance	Uncertain significance	–	1
p.Ala217Asp	ClinVar, MDSgene	Pathogenic	Pathogenic	Pathogenic	11.5	–
p.Met237Val	gnomAD	Uncertain significance	Uncertain significance	Uncertain significance	1.5	–
p.Glu240Lys	MDSgene, gnomAD	Pathogenic	Pathogenic	Pathogenic	6.5	–
p.Ala244Gly	MDSgene	Likely Pathogenic	Uncertain significance	Uncertain significance	2.5	–
p.Leu249Val	ClinVar, MDSgene, gnomAD	Uncertain significance	Uncertain significance	Uncertain significance	1	1
p.Thr257Ile	ClinVar, gnomAD	Uncertain significance	Uncertain significance	Uncertain significance	–	2
p.His271Gln	ClinVar, MDSgene	Pathogenic	Pathogenic	Pathogenic	5.5	–
p.Arg279His	ClinVar, MDSgene, gnomAD	Pathogenic	Uncertain significance	Uncertain significance	–	2
p.Pro296Leu	ClinVar, gnomAD	Uncertain significance	Uncertain significance	Uncertain significance	1.5	–
p.Gly309Asp	ClinVar, MDSgene	Pathogenic	Pathogenic	Pathogenic	5.5	–
p.Thr313Met	ClinVar, MDSgene, gnomAD	Pathogenic	Pathogenic	Pathogenic	14.5	–
p.Val317Ile	MDSgene, gnomAD	Likely Pathogenic	Uncertain significance	Uncertain significance	1.5	1
p.Cys323Tyr	gnomAD	Uncertain significance	Uncertain significance	Uncertain significance	0.5	1
p.Ala339Thr	ClinVar, MDSgene, gnomAD	Uncertain significance	Uncertain significance	Uncertain significance	1.5	1
p.Ala340Thr	ClinVar, gnomAD	Benign	Benign	Benign		7
p.Leu347Pro	ClinVar, gnomAD	Pathogenic	Pathogenic	Pathogenic	15.5	–
p.His360Arg	MDSgene	Likely Pathogenic	Uncertain significance	Uncertain significance	2.5	–
p.Asn367Ser	MDSgene, gnomAD	Likely Pathogenic	Uncertain significance	Uncertain significance	2	–
p.Ile368Asn	MDSgene, gnomAD	Likely Pathogenic	Uncertain significance	Pathogenic	5	–
p.Leu369Pro	MDSgene, gnomAD	Likely Pathogenic	Uncertain significance	Likely Pathogenic	4	–
p.Gly386Ala	MDSgene	Pathogenic	Pathogenic	Pathogenic	5.5	–
p.Cys388Arg	ClinVar, MDSgene	Likely Pathogenic	Pathogenic	Pathogenic	11.5	–
p.Gly409Arg	MDSgene, gnomAD	Likely Pathogenic	Likely pathogenic	Pathogenic	5.5	–
p.Gly409Val	MDSgene	Pathogenic	Pathogenic	Pathogenic	5.5	–
p.Pro416Leu	MDSgene	Pathogenic	Uncertain significance	Pathogenic	5.5	–
p.Pro416Arg	MDSgene	Pathogenic	Pathogenic	Pathogenic	7	–
p.Glu417Gly	MDSgene	Pathogenic	Pathogenic	Pathogenic	5.5	–
p.Ser419Pro	MDSgene	Likely Pathogenic	Likely pathogenic	Pathogenic	5	–
p.Trp437Arg	MDSgene	Likely Pathogenic	Uncertain significance	Pathogenic	5.5	–
p.Arg464His	MDSgene, gnomAD	Likely Pathogenic	Uncertain significance	Uncertain significance	3	–
p.Glu476Lys	ClinVar	Benign	Benign	Benign	–	9.5
p.Leu489Pro	MDSgene	Pathogenic	Pathogenic	Pathogenic	5	–
p.Arg501Gln	ClinVar, gnomAD	Benign	Benign	Benign	–	9.5
p.Asn521Thr	ClinVar, gnomAD	Likely benign	Benign	Benign	–	7
p.Ala537Thr	gnomAD	Uncertain significance	Uncertain significance	Uncertain significance	0.5	2.5

## Discussion

In this study, we systematically investigated EOPD-associated or population-based *PINK1* missense variants. Using the Sherloc framework, supplemented with cellular assays, we investigated 50 missense variants in *PINK1* that were mostly of *uncertain significance*. We found that 20 of the 46 patient-associated variants had sufficient evidence for a *pathogenic* annotation and were seldom found in the general population. Most of these *pathogenic* variants completely abolished mitophagy function and Parkin translocation. We also identified multiple missense variants that displayed intact mitophagy function but aberrant Parkin accumulation. Although our study provides insights into the functional consequences of the missense variants, interpretation of the pathological consequences remains challenging without also assessing clinical and population-based data with a comprehensive scoring system such as Sherloc. Therefore, we advise genetic diagnostic laboratories to acquire multiple layers of evidence including available functional data, to determine whether a variant truly is disease-causing or not and plea for the implementation of functional tests in the routine workflow of genetic diagnostic laboratories.

Even with the variant classification guidelines formulated by ACMG-AMP^8^, variant classification seems subject to personal interpretation^10^, especially for infrequently encountered variants. Our work shows that the clinical significance of many of the variants reported in the ClinVar and MDSgene patient databases were assumed to be more damaging than the evidence implied, as we have reclassified 11/46 variants reported *as (likely) pathogenic* to *uncertain significance*. However, using Sherloc, the classification of variants that were *(likely) pathogenic* was supported by multiple lines of evidence, such as a very low MAF, absence of homozygosity in population databases, segregation with disease and previously reported functional aberrations in different cellular assays.

With our systematic analysis of 50 *PINK1* missense variants for mitophagy and Parkin translocation defects, we have shown that most variants classified as *(likely) pathogenic* by Sherloc caused loss of PINK’s downstream functionality, as reflected by our observations of no or significantly reduced mitophagy induction and/or Parkin translocation. This is consistent with the idea that disease-causing *PINK1* variants confer pathogenicity through a loss-of-function mechanism leading to defects in PINK1 kinase activity^2,3^.

Not surprisingly, most *(likely) pathogenic* missense variants are localized in domains that are crucial for the kinase activity of PINK1. For instance, the PINK1 variants p.Ala168Pro, p.Val170Gly, p.Ala217Asp, and p.Leu369Pro, which were previously predicted to disrupt ATP-binding and kinase activity^20^, showed no mitophagy induction and/or Parkin recruitment in our functional studies. Likewise, the *pathogenic*PINK1 variant p.Thr313Met, which affects a phosphorylation site of PINK1 that is required for proper PINK1 activity^24^, showed significantly reduced mitophagy induction and loss of Parkin recruitment. Moreover, multiple *pathogenic* PINK1 missense variants that were previously predicted to change the protein fold of the kinase core (including p.Leu347Pro, p.Ile368Asn, p.Trp437Arg, and p.Leu489Pro) or the activation loop (including p.Gly386Ala, p.Gly388Arg, p.Gly409Arg, p.Gly409Val, p.Pro416Arg, and p.Pro416Leu)^20^ also showed abolished mitophagy function and Parkin recruitment. This data reinforces the idea that *pathogenic PINK1* variants cause loss of PINK1 kinase activity.

However, not all *(likely) pathogenic* variants exhibit loss or reduced PINK1-induced mitophagy and Parkin recruitment, e.g., variants p.His271Gln, p.Gly309Asp, p.Glu417Gly, and p.Ser419Pro. Intriguingly, these and other variants of *uncertain significance* had aberrant Parkin localization in the form of diffuse punctate aggregates instead of the perinuclear clustering observed for WT PINK1. Perinuclear clustering of mitochondria occurs as a result of Parkin-mediated K63-linked polyubiquitination of mitochondrial substrates, including p62^25–27^. This can only ensue with the precise interplay between ubiquitin, PINK1, and Parkin. In agreement with our study, others have shown that several patient-associated variants in Parkin^25^ and PINK1^26^ are unable to cluster mitochondria upon CCCP treatment. Therefore, we hypothesize that the missense variants that display diffuse punctate Parkin localization disturb the polyubiquitination mechanism. Of note are *PINK1* variants p.Pro296Leu and p.Gly309Asp that reside in Insertion 3, a crucial element of the kinase domain that is directly involved in ubiquitin binding and impair ubiquitin phosphorylation^20^. As phosphorylated ubiquitin facilitates the confirmational change of Parkin from inactive to active state^28^, these missense variants likely somewhat hinder the activation of Parkin. It is possible that similar obstructions occur with the other variants that aberrantly localize Parkin. In contrast, all *benign* PINK1 variants were able to accumulate Parkin in the perinuclear region. Notably, different studies have shown that mitochondrial clearance occurs despite p62-mediated translocation of mitochondria^25,27^, confirming our observations that the missense variants that affect Parkin translocation are able to induce mitophagy.

In addition, most PINK1 variants *of uncertain significance*, including those predicted to affect kinase function (p.Met237Val, p.Ala244Gly, p.Val317Ile, and p.Ala339Thr)^20^, did not cause complete loss of mitophagy and were able to activate Parkin. This illustrates that a change in the structure that is predicted to be damaging does not abolish kinase function per se. Furthermore, although all *benign* PINK1 variants were able to induce mitophagy, three *benign* PINK1 variants with relatively high MAF, p.Gln115Leu, p.Ala340Thr, and p.Asn521Thr, were less able to induce mitophagy compared to WT PINK1. This may suggest that some degree of defective mitophagy is tolerated in cells before pathogenic effects occur. Likewise, similarly reduced mitophagy induction was observed for some PINK1 variants of *uncertain significance*. While one could argue that these variants with seemingly similar functional effects, as *benign* variants, should be regarded as not disease-causing, we cannot exclude that a mitophagy-unrelated disease-causing mechanism may be conferred via other downstream PINK1 pathways, especially for those variants with aberrant Parkin translocation. Indeed, additional PINK1-Parkin‒mediated pathomechanisms for EOPD have been proposed^14,29^. Next to mitophagy, PINK1 and Parkin regulate a plethora of different mitochondrial functions^14,30^, such as mitochondrial dynamics^31–33^, transport of mitochondria^34,35^, biogenesis of mitochondria^36,37^ and formation of mitochondrial-derived vesicles^38^. Furthermore, recent studies highlight a key role for PINK1 as a repressor of the immune system through the repression of mitochondrial antigen presentation^39,40^. By studying the two most robust pathways—mitophagy and Parkin recruitment—reported to be linked to PINK1’s actions, we may have missed the effects of PINK1 variants that impact other functions. For example, the PINK1 variants p.Gly309Asp and p.Leu347Pro are associated with perturbed mitochondrial dynamics^41^ (see Supplementary Table 2 for more examples). Yet, they also showed impaired Parkin recruitment in our study, suggesting that Parkin may play a role in many of PINK1’s functions not related to mitophagy^14^. Additionally, it remains to be determined whether PINK1 variants with normal mitophagy and Parkin recruitment may confer pathogenicity through impairments in one of those functions not related to mitophagy and thus cannot yet be classified as *benign*. The variants that showed a clear loss-of-function in our functional assays, however, are expected to be disease-causing, which is also supported by other studies (Supplementary Table 2). An additional limitation in this study is the use of the immortalized HeLa cell line in conjunction with overexpression of PINK1 and Parkin to study the effects of the *PINK1* variants. As such, this artificial system differs considerably from the biological situation that causes disease and should be considered as a proxy for the real situation until it can be proven in models that more closely mimic the patient situation. Nonetheless, our approach allows for a practical investigation of the biochemical consequences of the missense variants that can be carried out in most laboratory settings. Finally, while it is possible to interrogate functional deficits for genes such as *PINK1* and *PRKN* that act in a mechanistic pathway that can be studied in a relatively straightforward manner, it remains difficult to do so for other genes linked to EOPD (e.g., *PARK7*) where the molecular function is less known.

Functional data, however, is only one line of evidence to evaluate pathogenicity. In order to make final decisions on the clinical significance of variants, functional studies should be supplemented by clinical data and evidence from population genetics. The *pathogenic* variants in our study could be assigned as such because of the extensive data availability, and this data was absent for most variants of *uncertain significance*. The presence of a missense variant in PINK1 in a Parkinson’s disease case does not automatically imply pathogenicity. The annotations from patient databases ClinVar and MDSgene should thus be interpreted with care, and we recommend using a consistent framework such as Sherloc for future annotations of variants in disease-linked genes such as *PINK1* found in patients.

## Methods

### Collecting *PINK1* variants

All the variants in the *PINK1* gene used in this study were collected from three publicly available databases (access date: 15 September 2020): MDSgene^5^, ClinVar^6^, and gnomAD version 2.1.1^7^. At the time of collection, the patient-specific Parkinson disease Mutation Database was unavailable^4^. Annotations about clinical significance were taken from ClinVar and MDSgene. Since each database used different classifications, we unified them into the standard annotation recommended by the ACMG-AMP (Supplementary Table 1).

### Annotation of *PINK1* variants using Sherloc

All variants were classified using the Sherloc framework, as previously described^10^. Briefly, pathogenic or benign points were assigned to each variant based on four layers of evidence: (1) population evidence, with pathogenic or benign points scored based on the MAF of variants in gnomAD^7^ or the dbSNP database^42^, (2) evidence from case reports (if available), where pathogenic points were given to rare (MAF < 8 counts with no homozygous variants) variants found in patients, (3) family-segregation information (if available), where pathogenic points were given for segregating variants, and (4) experimental evidence reported in the literature, where pathogenic, benign or conflicting points were scored for functional proof that a variant affected downstream function (e.g., Parkin recruitment or substrate phosphorylation). With respect to this last layer, we curated 54 studies that investigated at least one of the 50 variants in our study (see Supplementary Table 2 for overview of studies and their variants).

### Cell culture, transient transfections, and treatments

HeLa cells were maintained in Dulbecco’s Modified Eagle’s Medium (Invitrogen, Waltham, MA) supplemented with 10% fetal bovine serum (Invitrogen) and 1% Penicillin–Streptomycin (Gibco, Waltham, MA) in a 37 °C incubator with 5% CO_2_. Cells were transiently transfected using polyethylenimine (Sigma-Aldrich, Saint Louis, MO) in a 1:5 ratio (DNA:polyethylenimine) for 48 h prior to performing the experiments. To induce mitochondrial depolarization, HeLa cells were treated with 10 µM CCCP (Sigma-Aldrich) for the indicated times, prior to cell harvesting or fixation. DMSO was used as a control treatment.

### Generation of stable reporter PINK1 knock-out cell lines

PINK1 KO cells were obtained by CRISPR-Cas9‒mediated genome editing in HeLa cells, as previously described^43^. Briefly, a 20-nt sgRNA sequence that targets exon 2 of the *PINK1* gene was cloned into the pSpCas9(BB)-2A-GFP (PX458) plasmid (a gift from Prof. Feng Zhang (Broad Institute, Cambridge, MA), Addgene plasmid #48138) using the *BbsI* restriction enzyme to form the targeting plasmid expressing Cas9-GFP. Following validation, the PX458-sgRNA plasmid was transfected into HeLa cells. GFP-positive cells were single-cell sorted 48 h post-transfection using an SH800S cell sorter (Sony Biotechnology, San Jose, CA) and grown in separate cultures that were subsequently screened for the presence of frameshift mutations leading to nonsense-mediated decay on both alleles. PINK1 KO was validated and confirmed using western blotting.

First, the cDNA of mt-mKeima and WT Parkin were obtained from the pCHAC-mt-mKeima^13^ (a gift from Prof. Richard Youle (National Institutes of Health, Bethesda, MD), Addgene plasmid #72342) and pEGFP-Parkin vectors^44^ (a gift from Prof. Edward Fon (McGill University, Montreal, Quebec, Canada), Addgene plasmid #45875), respectively. Second, the cDNA of mitochondria-targeting mKeima (mt-mKeima) and WT FLAG-Parkin were cloned into the pIRES vector (Clontech Laboratories, Mountain View, CA). mt-mKeima cDNA was cloned between restriction sites NheI and EcoRI and FLAG-tagged Parkin cDNA was cloned at restriction site NotI in multiple cloning sites 1 and 2, respectively.

To obtain stable PINK1 KO reporter cell lines for mKeima-FLAG-Parkin and EGFP-Parkin, PINK1 KO HeLa cells were transfected with either the mt-mKeima-FLAG-Parkin or pEGFP-Parkin plasmids. After 48 h, the growth medium was replaced with selection medium containing 800 ng/µl G-418 (Sigma-Aldrich), which was refreshed every 3 days for 14 days. Single, stably expressing cells were sorted using an SH800S cell sorter (Sony Biotechnology). These single cells were grown into separate cell lines that were used in the following experiments.

### Cloning and site-directed mutagenesis of *PINK1*

To assess PINK1 function based on downstream functional consequences, we generated PINK1-HA expression vector by subcloning the pcDNA-DEST47 *PINK1* C-GFP^45^ (a gift from Prof. Mark Cookson (National Institutes of Health, Bethesda, MD), Addgene plasmid #13316) into the pcDNA3.1(−) vector (Thermo Fischer Scientific, Waltham, MA) between the restriction sites EcoRI and HindIII. To introduce the 50 missense variants into the cDNA of *PINK1*, site-directed mutagenesis was performed using the QuickChange II XL kit following the manufacturer’s protocol (Agilent, Santa Clara, CA). The complete cDNA of *PINK1* was subsequently analyzed using Sanger sequencing to validate the presence of the correct nucleotide change, as well as the absence of any other variants. The complete list of mutagenesis primers can be found in Supplementary Table 3.

### FACS analysis of mitophagy

HeLa PINK1 KO cells stably expressing mt-mKeima-FLAG-Parkin were transiently transfected with WT-PINK1-HA or variant-PINK1-HA. Cells were treated with either DMSO or 10 µM CCCP (Sigma-Aldrich) for 24 h, after which the cells were rinsed, dissociated with 0.05% Trypsin-EDTA (Thermo Fischer Scientific), resuspended in 4′,6-diamidino-2-phenylindole (DAPI)-containing (100 ng/ml) medium and transferred to FACS tubes. FACS analysis was performed with a Novocyte Quanteon flow cytometer (Agilent). mt-mKeima was detected using 488 and 561 nm lasers with a 615 nm emission filter to quantify the mitochondria present in the cytosol or lysosome, respectively. At least 50,000 events were recorded for each experiment, and each event was gated for a cell that was DAPI-negative and mt-mKeima-positive. The population of cells with a high 561:488 nm ratio was determined by drawing a gate in the upper left quadrant of DMSO-treated untransfected cells, which was subsequently used for all samples in the same independent experiment. Mitophagy induction was quantified as the increase in this cell population upon CCCP treatment for each PINK1 variant minus the control condition (untreated cells) and normalized against cells expressing WT-PINK1-HA from the same experiment. In addition, MFI was determined for the gated cells, using a high 561:488 nm ratio as a second measure of the amount of mitophagy induction. Background MFI was determined by the MFI from the population of non-induced mKeima-positive cells (low 561:488 ratio) and was subtracted from the MFI of the induced mKeima-positive cells and then normalized against WT-PINK1-HA from the same independent experiment. Data analysis was performed using Kaluza Analysis software (Beckman Coulter, Brea, CA).

### EGFP-Parkin localization using immunofluorescence

HeLa cells stably expressing EGFP-Parkin were seeded on glass coverslips in 24-well plates and transiently transfected with WT-PINK1-HA or variant-PINK1-HA. Subsequently, cells were treated with DMSO or 10 µM CCCP (Sigma-Aldrich) for 6 h following fixation in 4% paraformaldehyde in PBS for 10 min at room temperature. Cells were then permeabilized in 0.1% Triton X-100 in PBS for 10 min and blocked with 3% bovine serum albumin in PBS for 1 h. Coverslips were then incubated overnight at 4 °C with rat anti-HA IgG monoclonal antibody (3F10, Roche, Basel, Switzerland; 1:250), washed, and subsequently incubated for 1 h at room temperature with goat anti-Rat IgG (H+L) Alexa Fluor 594 secondary antibody (Invitrogen; 1:250). Coverslips were mounted onto glass slides in DAPI-containing mounting medium (Vector Laboratories, Burlingame, CA). The slides were analyzed using structured illumination microscopy (SIM). SIM images were acquired with an AxioObserver Z1 compound microscope (Carl Zeiss, Oberkochen, Germany) equipped with an Apotome, 63x oil objective, and an AxioCam MRm3 CCD camera (Carl Zeiss). Images were captured for each condition with identical exposure times. Translocation of EGFP-Parkin was assessed in cells that expressed both WT- or variant-PINK1-HA and EGFP-Parkin and scored by a blinded observer for either diffuse or aggregated EGFP-Parkin, as previously described^44^. At least 75 cells were scored per variant in three independent experiments.

### Protein extraction and immunoblotting

Cells were harvested in 2% sodium dodecyl sulfate (SDS)/phosphate-buffered saline (PBS) buffer containing a proteinase inhibitor cocktail (Roche) and sonicated. Protein concentrations were quantified using the Pierce^TM^ BCA protein assay kit (Thermo Fischer Scientific), and samples were mixed with loading sample buffer containing 10% β-mercaptoethanol before being boiled at 95 °C for 5 min. Subsequently, equal amounts of total protein extracts were subjected to SDS-PAGE, transferred to nitrocellulose membranes, blocked for 1 h in skimmed milk, and incubated overnight with primary antibody at 4 °C and then with for the corresponding secondary antibody for 1 h at room temperature. Blots were imaged on a ChemidocTM MP Imaging System (Bio-Rad, Hercules, CA). All blots were derived from the same experiment and were processed in parallel. Original uncut blots are shown in Supplementary Fig. 8. The primary antibodies used were mouse anti-β-Actin (MP Biomedicals 8691001, 1:5000), mouse anti-Parkin (Santa-Cruz Biotechnology, Dallas, TX, sc-32282, 1:500), and rabbit anti-PINK1 (D8G3, Cell signaling, Danvers, MA,1:1000). The secondary antibodies were HRP-conjugated goat anti-mouse IgG (H + L) (Bio-Rad, 1:10,000) and HRP-conjugated goat anti-rabbit IgG (H + L) (Bio-Rad, 1:10,000).

### Statistical analyses

The data obtained from the FACS and Parkin localization experiments were analyzed using a linear model by one-way analysis of variance (ANOVA) followed by Tukey’s post-hoc test. Data are means ± standard error of the mean from at least three independent experiments. A *p*-value < 0.05 was considered statistically significant. Statistical analyses were computed in R (version 1.3.959).

### Reporting summary

Further information on research design is available in the Nature Research Reporting Summary linked to this article.

## Supplementary information

Supplementary Information

Reporting Summary

## Data Availability

The raw data and fluorescence images are available from the corresponding authors upon reasonable request.
