# Supplementary material for: Systematic analysis of PINK1 variants of unknown significance shows intact mitophagy function for most variants
Source: NPJ Parkinsons Dis. 2021 Dec 10;7:113. doi: 10.1038/s41531-021-00258-8 (PMC8664852; doi:10.1038/s41531-021-00258-8)
Supplement: Supplementary file 1 — Supplementary Information [file 41531_2021_258_MOESM1_ESM.pdf]

a

Evidence of pathogenicity	Category
Very Strong	PVS1: Null variant (nonsense, frameshift, canonical 1 or 2 splice sites, initiation codon, single or multi-exon deletion) in a gene where loss of function is a known mechanism of disease
Strong	PS3: Well-established *in vitro* or *in vivo* functional studies supportive of a damaging effect on the gene or gene product
Moderate	PS4 (counts as moderate for rare recessive disorders): The prevalence of the variant in affected individuals is significantly increased compared with the prevalence in controls
	PM2: Absent from controls (or at extremely low frequency if recessive) in Genome Aggregation Database
	PM3: For recessive disorders, detected in trans with a pathogenic variant
	PM5: Novel missense change at amino acid residue where a different missense change determined to be pathogenic has been seen before
Supporting	PP1: Co-segregation with disease in multiple affected family members in a gene definitively known to cause the disease
	PP3: Multiple lines of computational evidence support a deleterious effect on the gene or gene product (conservation, evolutionary, splicing impact, etc.)

b

Evidence of benign impact	Category
Stand-alone	BA1: Allele frequency is >5% in Exome Sequencing Project, 1000 Genomes Project, or Exome Aggregate Consortium
Strong	BS1: Allele frequency is greater than expected for disorder
	BS2: Observed in a healthy adult individual for a recessive (homozygous), dominant (heterozygous), or X-linked (hemizygous) disorder, with full penetrance expected at early age
	BS3: Well-established *in vitro* or *in vivo* functional studies show no damaging effect on protein function or splicing
	BS4: Lack of segregation in affected members of family
Supporting	BP2: Observed in *trans* with a pathogenic variant for a fully penetrant dominant gene/disorder or observed in *cis* with a pathogenic variant in any inheritance pattern
	BP4: Multiple lines of computational evidence suggest no impact on gene or gene product (conservation, evolutionary, splicing impact, etc.)

c

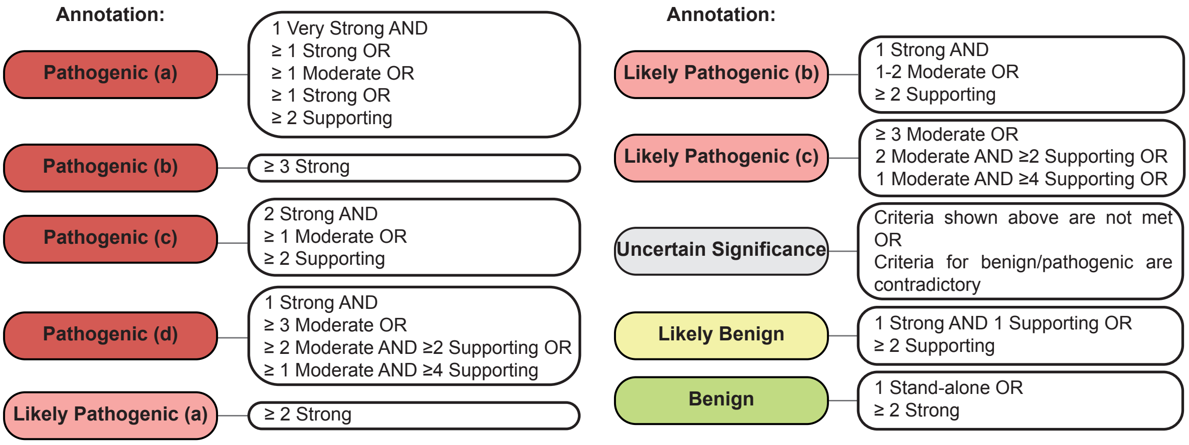

Supplementary Figure 1. Overview of relevant ACMG-AMP guidelines for missense variant interpretation **a)** Criteria for the evaluation of different levels of pathogenic evidence. Very strong evidence (PVS1) is not applicable for missense variants. **b)** Criteria for the evaluation of different levels of benign evidence. **c)** Scheme for the assessment of pathogenicity based on the categories of pathogenic and benign criteria. This figure is adapted from the updated Best Practice guidelines of the Association for Clinical Genomic Science by Ellard et al[9].

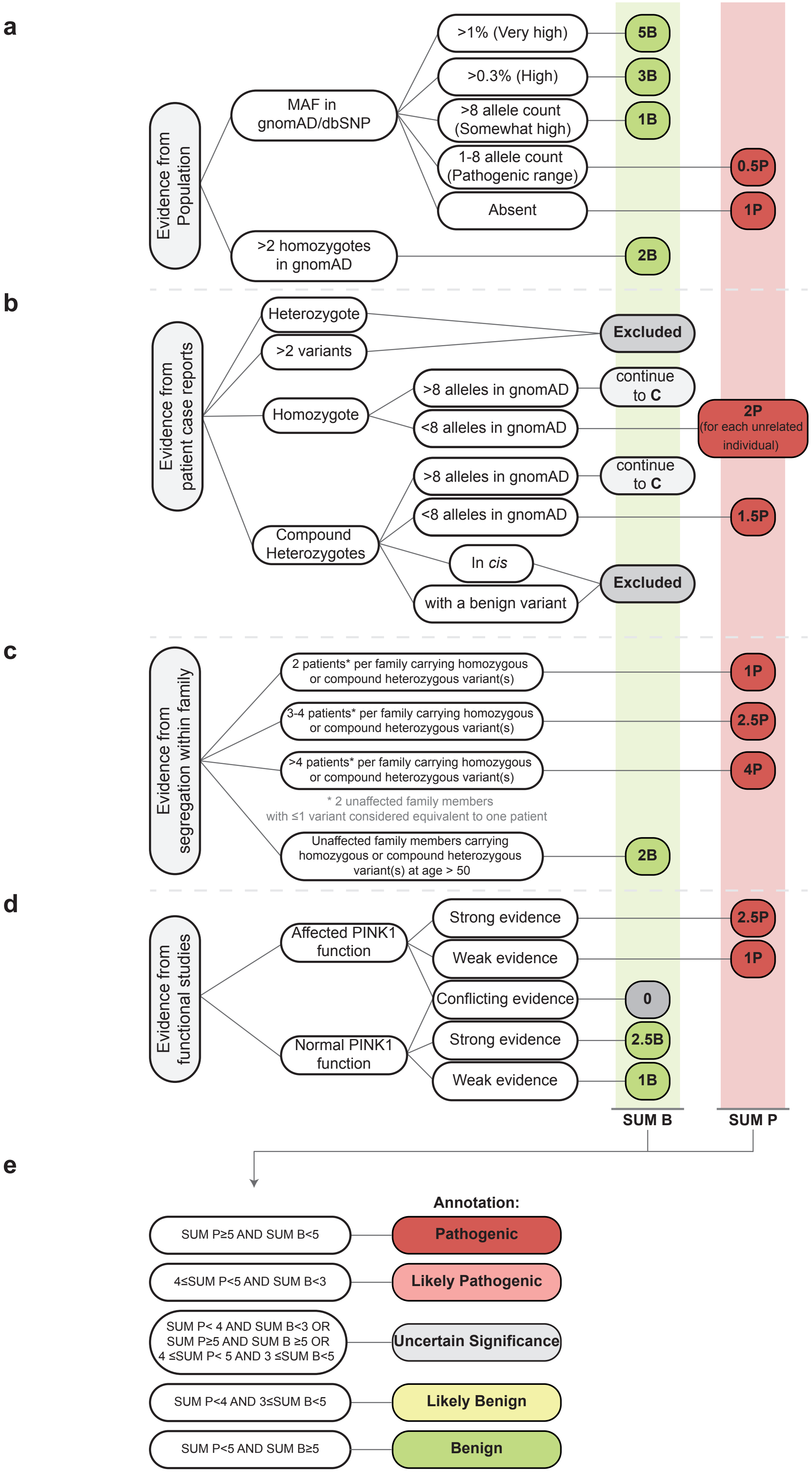

Supplementary Figure 2. Schematic overview of the scoring system of the Sherloc framework
The four layers of evidence were used to assign points: **a)** the MAF and homozygosity status of the variant in population-wide databases, **b)** the presence of rare variant in EOPD case reports, **c)** segregation of the variant with EOPD within a family, and **d)** functional proof of deleterious effects on PINK1 function. Points were then combined and clinical annotations were given as shown in **(e)**.

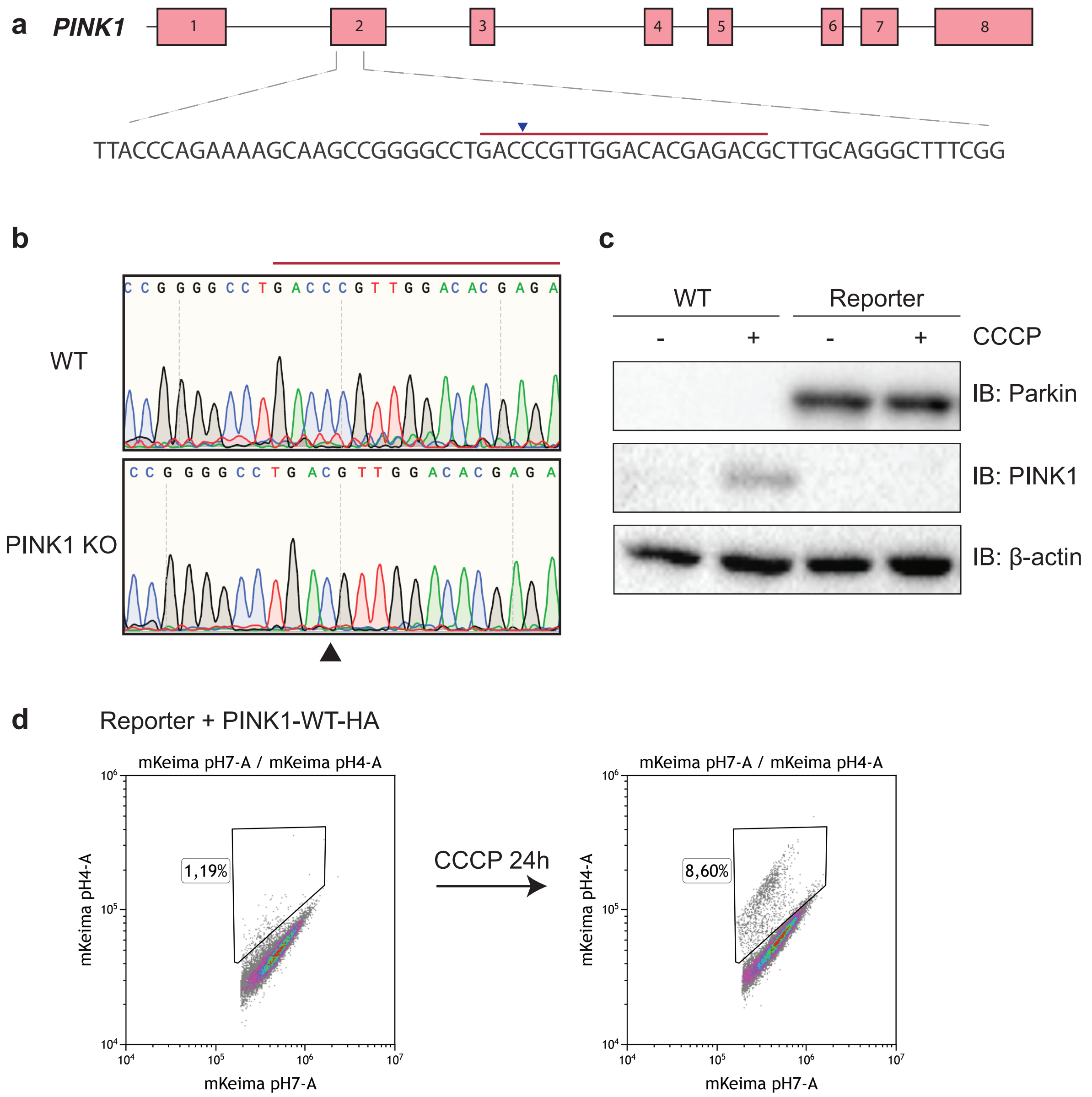

Supplementary Figure 3. Generation of HeLa PINK1 KO stably expressing mt-mKeima-FLAG-Parkin
a) Schematic representation of the strategy for CRISPR-Cas9 editing of the PINK1 gene. Partial sequence of target on exon 2 is shown, including the 20-nt guide sequence depicted by the burgundy line with the blue arrowhead pointing towards the predicted Cas9-cleavage site. **b)** Sanger chromatograms of the region surrounding the CRISPR-edit of WT HeLa cells (upper panel) and the HeLa KO clone used in this study (lower panel). The 20-nt guide sequence is depicted by the burgundy line. Black arrowhead indicates a homozygous CC-deletion that is predicted to cause a frameshift and premature termination of PINK1 mRNA. **c)** Representative immunoblots of protein extracts from WT HeLa and HeLa PINK1 KO mt-mKeima-FLAG-Parkin (reporter) cells treated with DMSO or CCCP for 6 h. Blots were stained for Parkin, PINK1 and β-actin (total protein loading control) antibodies. **d)** Scatter plots of FACS analysis of PINK1 WT-expressing HeLa PINK1 KO cells stably expressing mt-mKeima-FLAG-Parkin upon DMSO (left) or CCCP treatment (right). The gate with high 561:488 nm ratio mt-mKeima used to quantify mitophagy induction is shown.

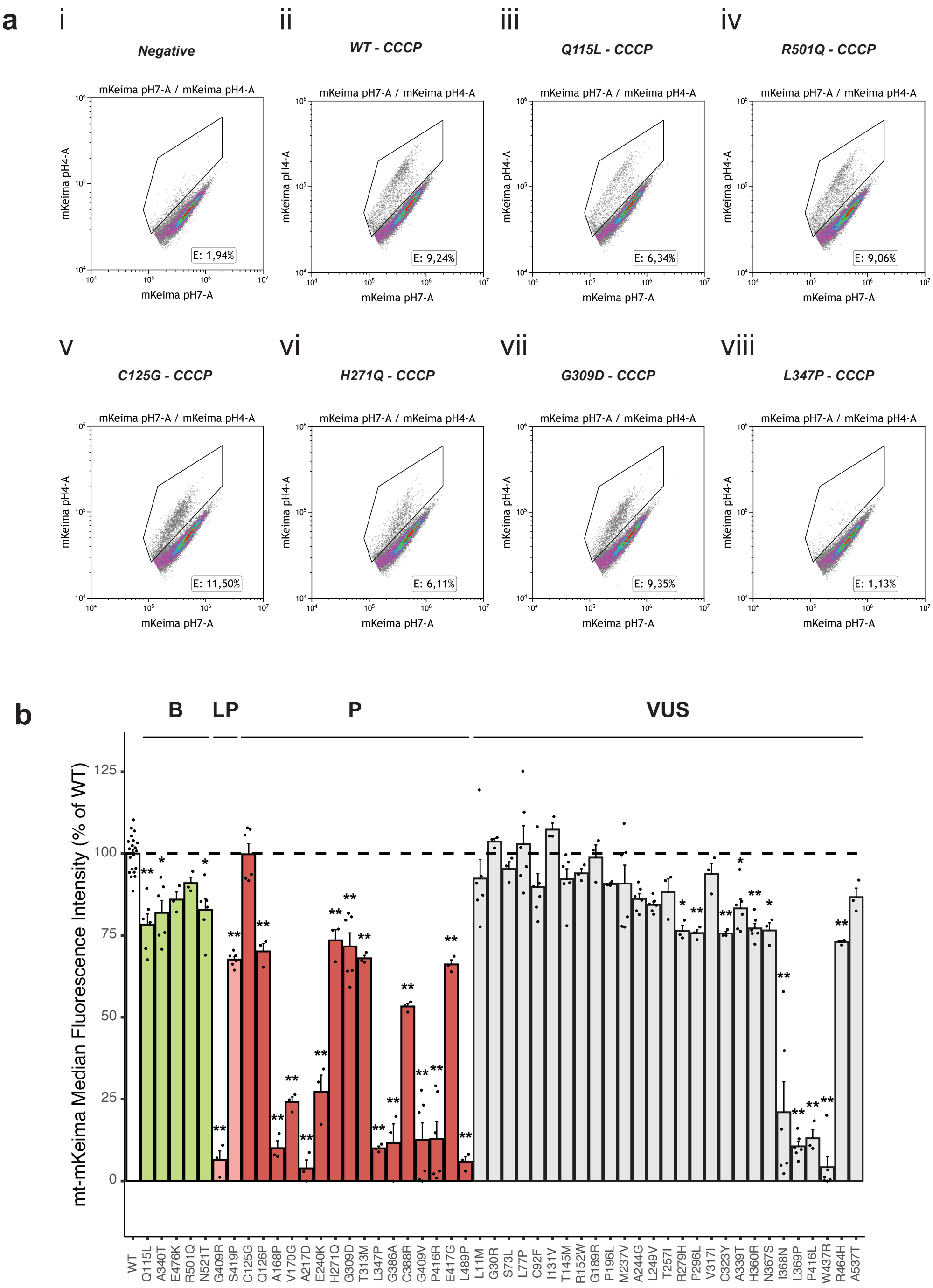

Supplementary Figure 4. Mitophagy analysis using the mt-mKeima reporter

a) Scatter plots of FACS analysis of PINK1 WT- or variant-expressing HeLa PINK1 KO cells stably expressing mt-mKeima-FLAG-Parkin upon DMSO or CCCP treatment. The gate with high 561:488 nm ratio mt-mKeima used to quantify mitophagy induction is shown. **b)** Assessment of median fluorescence intensity of mt-mKeima in the gate shown in (A) upon CCCP treatment in HeLa PINK1 KO cells stably expressing mt-mKeima-FLAG-Parkin transfected with by PINK1 WT or variants. All data shown is background-corrected and normalized against PINK1 WT control. Each dot represents a separate experiment. Bars are color-coded by the annotation determined by Sherloc. Data analyzed using one-way ANOVA. * $p < 0.05$, ** $p < 0.005$.

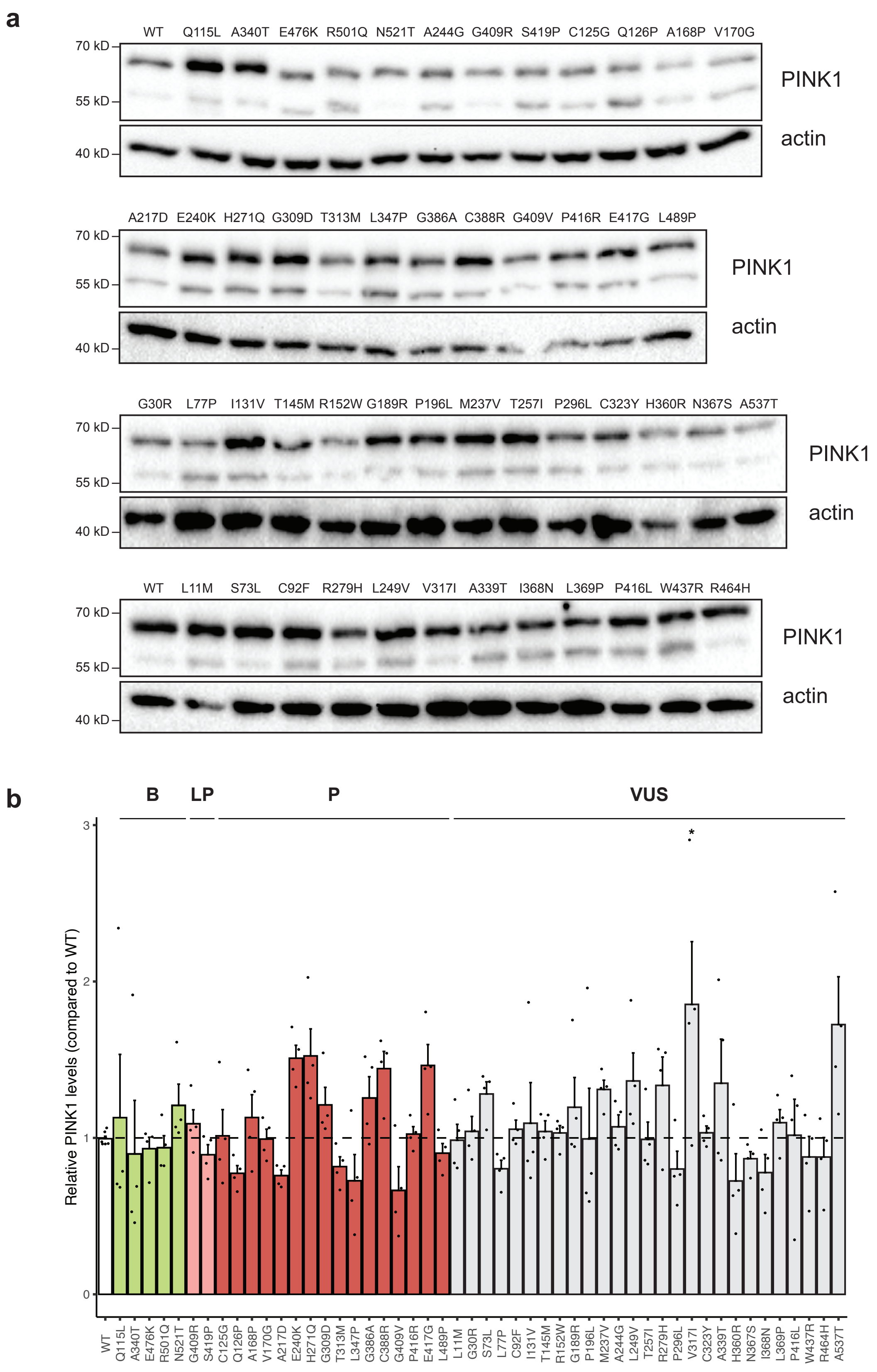

Supplementary Figure 5. All PINK1 missense variants are expressed after transfection
a) Representative immunoblots of protein extracts from WT HeLa and HeLa PINK1 KO mt-mKeima-FLAG-Parkin (reporter) cells transfected with WT or PINK1 missense variants. Blots were stained for PINK1 and β-actin (total protein loading control) antibodies. **b)** Quantification of PINK1 protein expression after transfection. Each dot represents a separate experiment. Bars are color-coded by the annotation determined by Sherloc. Data analyzed using one-way ANOVA. * $p < 0.05$

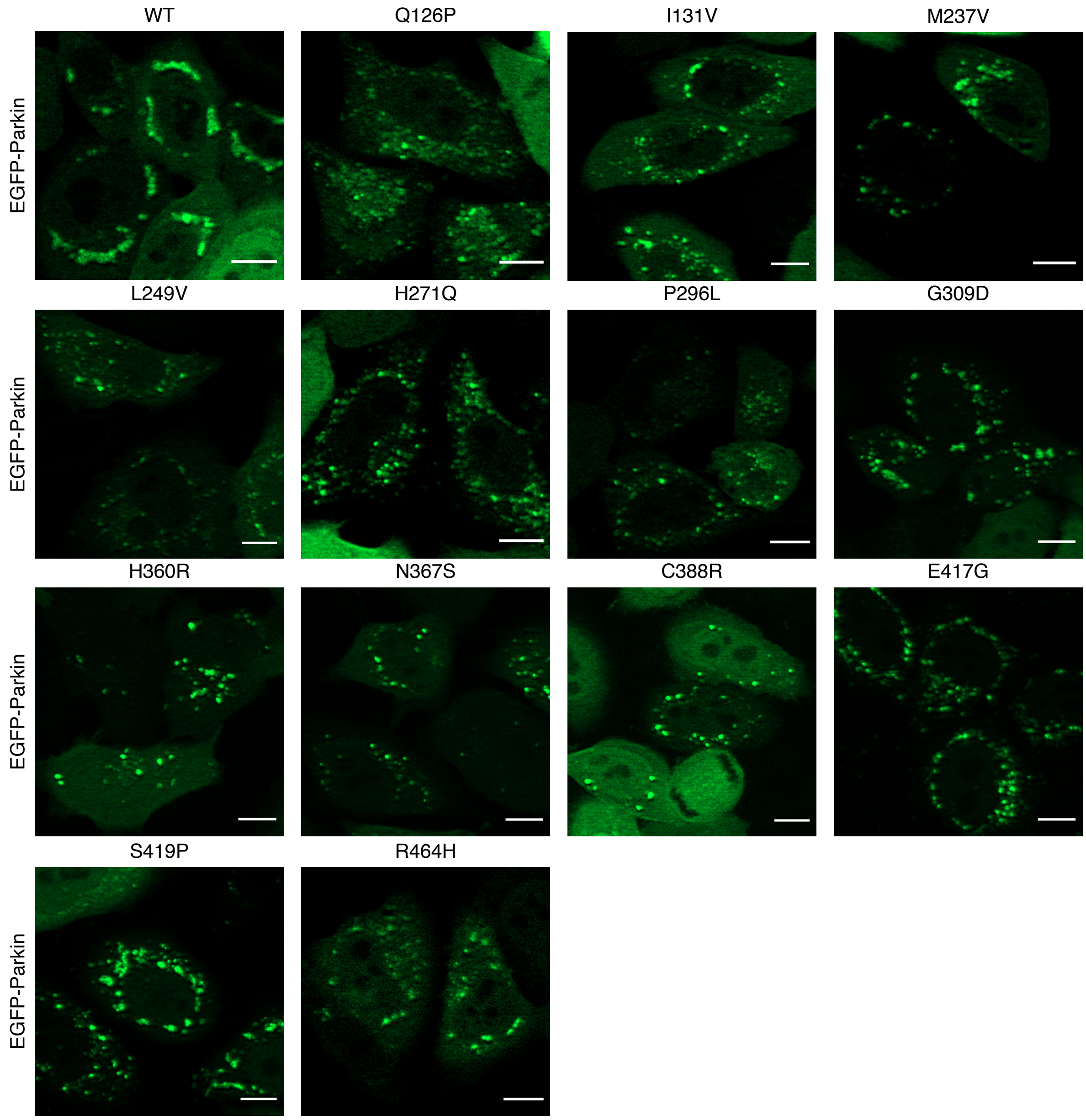

Supplementary Figure 6. Several PINK1 missense variants cause an aberrant Parkin translocation into diffuse punctate aggregates

Representative fluorescence images of HeLa PINK1 KO cells stably expressing EGFP-Parkin transfected with PINK1 WT and PINK1 variants after treatment with CCCP showing punctate diffuse aggregates of EGFP-Parkin. Scale bar, 10 μm

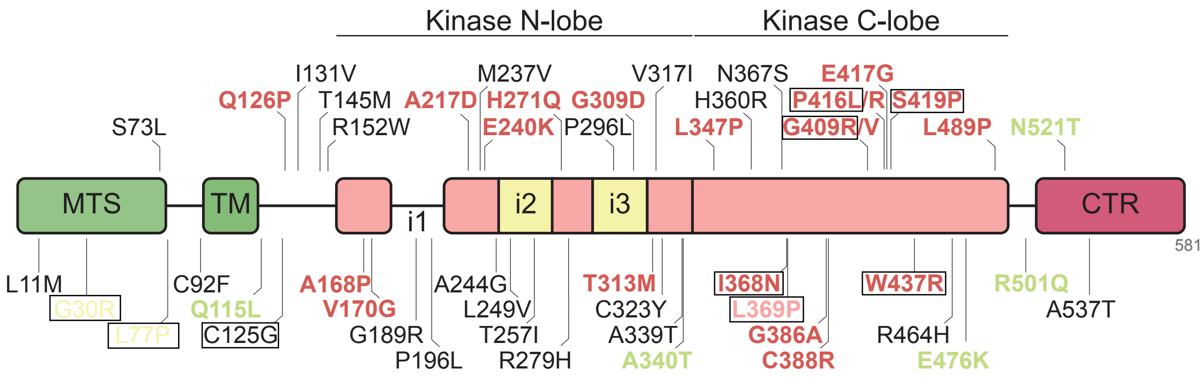

Supplementary Figure 7. Overview of updated annotations of PINK1 missense variants used in this study
Updated schematic overview of the functional domains of PINK1 with the location of the 50 missense variants. Variants are color-coded based on their updated classification. MTS = mitochondrial targeting sequence; TM = transmembrane domain; CTR = C-terminal region.

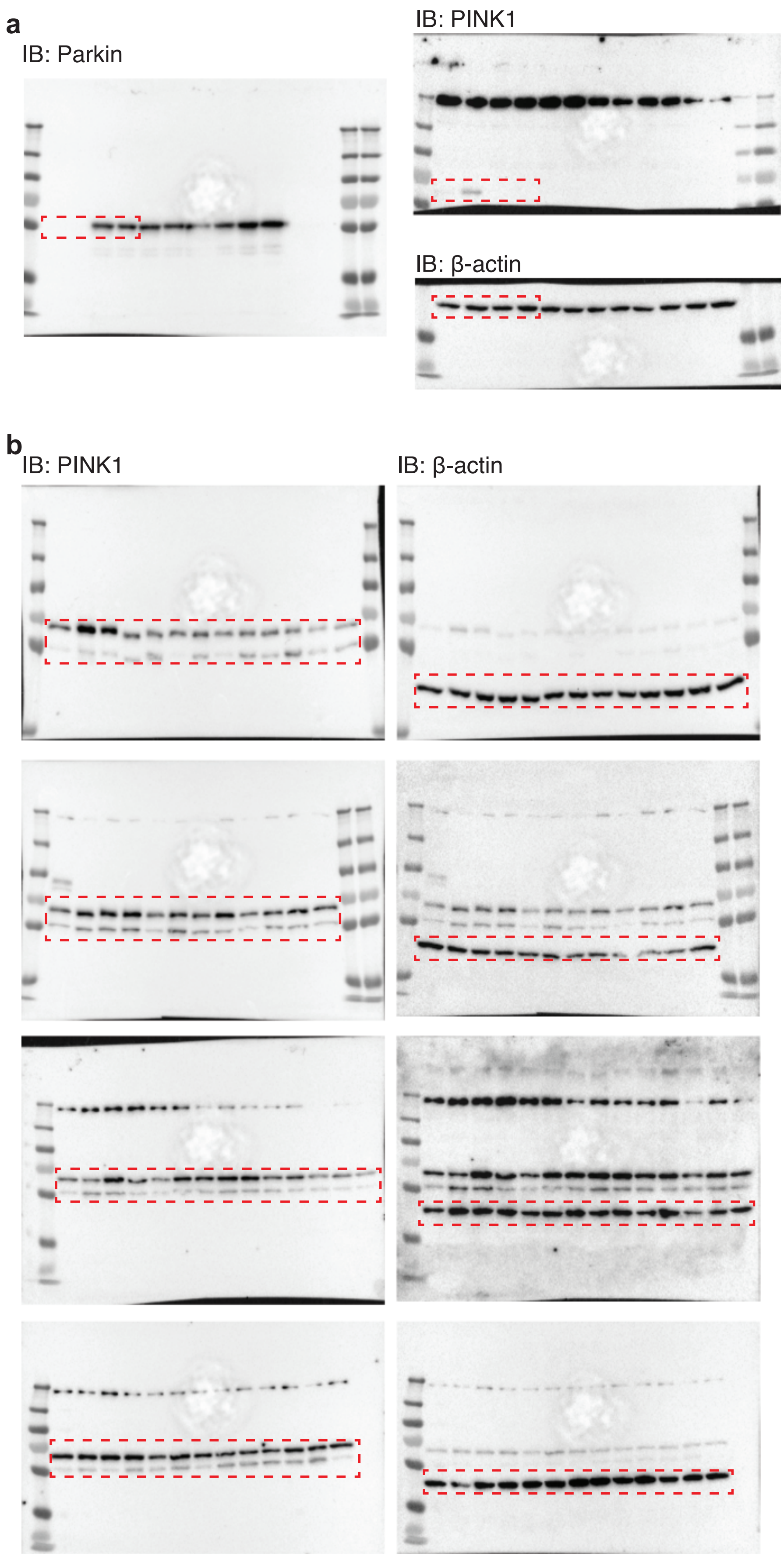

Supplementary Figure 8. Original uncut western blots
a) Original blots shown in Supplementary Figure 3c. b) Original blots shown in Supplementary Figure 5a

Supplementary Table 1. Complete overview of *PINK1* variants used in the study

Databases	Databases	Databases	cDNA variant	PINK1 variant	Abbreviation	Amino Acid codon	Clinical significance ClinVar	Clinical significance MDSGene	Combined annotation (ClinVar + MDSgene)	dbSNP ID	gnomAD allele count	Zygosity
	ClinVar		c.31C>A	p.Leu11Met	L11M	11	Uncertain significance		Uncertain significance	rs886044686	0	Homozygous
	ClinVar	GnoMAD	c.88G>C	p.Gly30Arg	G30R	30	Conflicting interpretations of pathogenicity		Uncertain significance	rs569753606	94	Homozygous
MDSGene	ClinVar	GnoMAD	c.218C>T	p.Ser73Leu	S73L	73	Uncertain significance	Probably pathogenic	Uncertain significance	rs202048763	43	Heterozygous *trans*
MDSGene			c.230T>C	p.Leu77Pro	L77P	77		Probably pathogenic	Likely Pathogenic		0	Heterozygous *trans*
MDSGene			c.275G>T	p.Cys92Phe	C92F	92		Probably pathogenic	Likely Pathogenic		0	Heterozygous *trans*
	ClinVar	GnoMAD	c.344A>T	p.Gln115Leu	Q115L	115	Benign		Benign	rs148871409	7378	Homozygous
MDSGene			c.373T>G	p.Cys125Gly	C125G	125		Probably pathogenic	Likely Pathogenic		0	Heterozygous *trans*
MDSGene		GnoMAD	c.377A>C	p.Gln126Pro	Q126P	126		Probably pathogenic	Likely Pathogenic		1	Homozygous
		GnoMAD	c.398A>G	p.Ile131Val	I131V	131			Uncertain significance	rs780200003	6	-
	ClinVar	GnoMAD	c.434C>T	p.Thr145Met	T145M	145	Conflicting interpretations of pathogenicity		Uncertain significance	rs45604240	8	Homozygous
	ClinVar	GnoMAD	c.454C>T	p.Arg152Trp	R152W	152	Uncertain significance		Uncertain significance	rs45608139	7	Heterozygous *trans*
MDSGene	ClinVar	GnoMAD	c.502G>C	p.Ala168Pro	A168P	168	Pathogenic	Definitely pathogenic	Pathogenic	rs768091663	5	Homozygous
MDSGene			c.509T>G	p.Val170Gly	V170G	170		Definitely pathogenic	Pathogenic		0	Homozygous
	ClinVar	GnoMAD	c.565G>A	p.Gly189Arg	G189R	189	Uncertain significance		Uncertain significance	rs757581951	15	Homozygous
	ClinVar	GnoMAD	c.587C>T	p.Pro196Leu	P196L	196	Uncertain significance		Uncertain significance	rs138302371	51	Homozygous
MDSGene	ClinVar		c.650C>A	p.Ala217Asp	A217D	217	Pathogenic	Probably pathogenic	Pathogenic		0	Homozygous
		GnoMAD	c.709A>G	p.Met237Val	M237V	237			Uncertain significance	rs775165635	3	Homozygous
MDSGene		GnoMAD	c.718G>A	p.Glu240Lys	E240K	240		Definitely pathogenic	Pathogenic	rs573931674	1	Homozygous
MDSGene			c.731C>G	p.Ala244Gly	A244G	244		Probably pathogenic	Likely Pathogenic		0	Heterozygous *trans*
MDSGene	ClinVar	GnoMAD	c.745T>G	p.Leu249Val	L249V	249	Uncertain significance	Possibly pathogenic	Uncertain significance	rs145650643	25	Heterozygous *trans*
	ClinVar	GnoMAD	c.770C>T	p.Thr257Ile	T257I	257	Uncertain significance		Uncertain significance	rs370906995	19	Homozygous
MDSGene	ClinVar		c.813C>A	p.His271Gln	H271Q	271	Pathogenic	Definitely pathogenic	Pathogenic	rs28940284	0	Homozygous
MDSGene	ClinVar	GnoMAD	c.836G>A	p.Arg279His	R279H	279	Pathogenic	Possibly pathogenic	Pathogenic	rs74315358	12	Heterozygous *trans*
	ClinVar	GnoMAD	c.887C>T	p.Pro296Leu	P296L	296	Uncertain significance		Uncertain significance	rs779060308	2	Homozygous
MDSGene	ClinVar		c.926G>A	p.Gly309Asp	G309D	309	Pathogenic	Definitely pathogenic	Pathogenic	rs74315355	0	Homozygous

MDSGene	ClinVar	GnoMAD	c.938C>T	p.Thr313Met	T313M	313	Pathogenic	Probably pathogenic	Pathogenic	rs74315359	6	Homozygous
MDSGene		GnoMAD	c.949G>A	p.Val317Ile	V317I	317		Probably pathogenic	Likely Pathogenic	rs200949139	11	Heterozygous *trans*
		GnoMAD	c.967G>A	p.Cys323Tyr	C323Y	323			Uncertain significance	rs373417787	8	-
MDSGene	ClinVar	GnoMAD	c.1015G>A	p.Ala339Thr	A339T	339	Conflicting interpretations of pathogenicity	Probably pathogenic	Uncertain significance	rs55831733	163	Heterozygous *trans*
	ClinVar	GnoMAD	c.1018G>A	p.Ala340Thr	A340T	340	Benign		Benign	rs3738136	24907	Homozygous
MDSGene	ClinVar	GnoMAD	c.1040T>C	p.Leu347Pro	L347P	347	Pathogenic	Definitely pathogenic	Pathogenic	rs28940285	5	Homozygous
MDSGene			c.1079A>G	p.His360Arg	H360R	360		Probably pathogenic	Likely Pathogenic		0	Heterozygous *trans*
MDSGene		GnoMAD	c.1100A>G	p.Asn367Ser	N367S	367		Probably pathogenic	Likely Pathogenic	rs749040285	2	Heterozygous *trans*
MDSGene		GnoMAD	c.1103T>A	p.Ile368Asn	I368N	368		Probably pathogenic	Likely Pathogenic	rs774647122	1	Homozygous
MDSGene		GnoMAD	c.1106T>C	p.Leu369Pro	L369P	369		Probably pathogenic	Likely Pathogenic	rs1195888869	1	Heterozygous *trans*
MDSGene			c.1157G>C	p.Gly386Ala	G386A	386		Definitely pathogenic	Pathogenic		0	Homozygous
MDSGene	Clinvar		c.1162T>C	p.Cys388Arg	C388R	388	Likely pathogenic	Definitely pathogenic	Likely Pathogenic		0	Homozygous
MDSGene		GnoMAD	c.1225G>A	p.Gly409Arg	G409R	409		Probably pathogenic	Likely Pathogenic	rs574890623	2	Homozygous
MDSGene			c.1226G>T	p.Gly409Val	G409V	409		Definitely pathogenic	Pathogenic		0	Homozygous
MDSGene			c.1247C>T	p.Pro416Leu	P416L	416		Probably pathogenic	Pathogenic		0	Homozygous
MDSGene			c.1247C>G	p.Pro416Arg	P416R	416		Probably pathogenic	Pathogenic		0	Homozygous
MDSGene			c.1250A>G	p.Glu417Gly	E417G	417		Definitely pathogenic	Pathogenic		0	Homozygous
MDSGene			c.1255T>C	p.Ser419Pro	S419P	419		Probably pathogenic	Likely Pathogenic		0	Homozygous
MDSGene			c.1309T>C	p.Trp437Arg	W437R	437		Probably pathogenic	Likely Pathogenic		0	Homozygous
MDSGene		GnoMAD	c.1391G>A	p.Arg464His	R464H	464		Possibly pathogenic	Likely Pathogenic	rs764328076	6	Heterozygous *trans*
	ClinVar	GnoMAD	c.1426G>A	p.Glu476Lys	E476K	476	Benign		Benign	rs115477764	1118	Homozygous
MSDGene			c.1466T>C	p.Leu489Pro	L489P	489		Definitely pathogenic	Pathogenic		0	Heterozygous *trans*
	ClinVar	GnoMAD	c.1502G>A	p.Arg501Gln	R501Q	501	Benign		Benign	rs61744200	909	Homozygous
	ClinVar	GnoMAD	c.1562A>C	p.Asn521Thr	N521T	521	Benign/Likely benign		Likely benign	rs1043424	82471	Homozygous
		GnoMAD	c.1609G>A	p.Ala537Thr	A537T	537			Uncertain significance	rs771032673	8	-

Supplementary Table 1 continued.

PINK1 variant	A	B	C	D	SUM P	SUM B	Sherloc Clincal Significance	ACMG Best practice 2020 Evidence	ACMG Clinical Significance	D updated with assays	SUM P (updated)	SUM B (updated)	Sherloc + assays Clincal Significance
p.Leu11Met	1P				1		Uncertain significance	PM2	Uncertain significance	1B	1	1	Uncertain significance
p.Gly30Arg	3B					3	Uncertain significance	BS1	Uncertain significance	1B		4	Likely Benign
p.Ser73Leu	1B					1	Uncertain significance	PM3	Uncertain significance	2.5B		3.5	Likely Benign
p.Leu77Pro	1P	2P			3		Uncertain significance	PM2, PM3	Uncertain significance	1B	3	1	Uncertain significance
p.Cys92Phe	1P	1.5P		0	3.5		Uncertain significance	PM2, PM3	Uncertain significance	1B	3.5	1	Uncertain significance
p.Gln115Leu	2B +5B			0		7	Benign	BS1, BS2	Benign	1P		7	Benign
p.Cys125Gly	1P	1.5P		2.5P	5		Pathogenic	PS3, PM2, PM3, PP1	Likely pathogenic (b)	0	2.5		Uncertain significance
p.Gln126Pro	0.5P	2P+2P	2.5P; 2 sibs homo + 3 unaffected family members with ≤1 variant	2.5P	9.5		Pathogenic	PS3, PS4 (moderate), PM2, PP1	Likely pathogenic (b)	2.5P	9.5		Pathogenic
p.Ile131Val	0.5P				0.5		Uncertain significance	PM2	Uncertain significance	1P	0.5		Uncertain significance
p.Thr145Met	0.5P			2.5B	0.5	2.5	Uncertain significance	PM2, BS2, BS3	Uncertain significance	2.5B	0.5	2.5	Uncertain significance
p.Arg152Trp	0.5P	1.5P			2		Uncertain significance	PM2, PM3	Uncertain significance	2.5B	2	2.5	Uncertain significance
p.Ala168Pro	0.5P	2P+2P		2.5P	6.5		Pathogenic	PS3, PM2, PP1, PP3	Likely pathogenic (b)	2.5P	6.5		Pathogenic
p.Val170Gly	1P	2P		2.5P	5.5		Pathogenic	PS3, PM2, PP1, PP3	Likely pathogenic (b)	2.5P	5.5		Pathogenic
p.Gly189Arg	1B					1	Uncertain significance	PM2	Uncertain significance	2.5B		1	Uncertain significance
p.Pro196Leu	1B			1P	1	1	Uncertain significance	PS3	Uncertain significance	0		1	Uncertain significance
p.Ala217Asp	1P	2P+2P	4P; 5 sibs homo + 2 sibs homo	2.5P	11.5		Pathogenic	PS3, PS4 (moderate), PM2, PP3	Likely pathogenic (b)	2.5P	11.5		Pathogenic
p.Met237Val	0.5P						Uncertain significance	PM2, PP3	Uncertain significance	1P	1.5		Uncertain significance
p.Glu240Lys	0.5P	2P+1.5P		2.5P	6.5		Pathogenic	PM2, PP3	Uncertain significance	2.5P	6.5		Pathogenic
p.Ala244Gly	1P	1.5P		1P	3.5		Uncertain significance	PM2, PM3, PP3	Uncertain significance	0	2.5		Uncertain significance
p.Leu249Val	1B					1	Uncertain significance	PM3	Uncertain significance	1P	1	1	Uncertain significance
p.Thr257Ile	1B					1	Uncertain significance	PP3	Uncertain significance	1B		2	Uncertain significance
p.His271Gln	1P	2P		2.5P	5.5		Pathogenic	PS3, PM2, PP3	Likely pathogenic (b)	2.5P	5.5		Pathogenic
p.Arg279His	1B					1	Uncertain significance	PM2, PM3	Uncertain significance	1B		2	Uncertain significance
p.Pro296Leu	0.5P			0	0.5		Uncertain significance	PM2, PP3	Uncertain significance	1P	1.5		Uncertain significance
p.Gly309Asp	1P	2P	?	2.5P	5.5		Pathogenic	PS3, PM2, PP3	Likely pathogenic (b)	2.5P	5.5		Pathogenic

p.Thr313Met	0.5P	4*2P	2.5P; 3 sibs homo & 1P; sibs homo	2.5P	14.5		Pathogenic	PS3, PS4 (moderate), PM2, PP3	Likely pathogenic (b)	2.5P	14.5		Pathogenic
p.Val317Ile	1B	1.5P		1P	2.5	1	Uncertain significance	PS3, PM2, PM3, PP3	Likely pathogenic (b)	0	1.5	1	Uncertain significance
p.Cys323Tyr	0.5P				0.5		Uncertain significance	PM2, PP3	Uncertain significance	1B	0.5	1	Uncertain significance
p.Ala339Thr	1B	1.5P		1P	1.5	2	Uncertain significance	PS3, PM3, PP3	Likely pathogenic (b)	0	1.5	1	Uncertain significance
p.Ala340Thr	2B +5B			1B		8	Benign	BA1, BS1, BS3	Benign	0		7	Benign
p.Leu347Pro	0.5P	5*2P	2.5P; 3 sibs homo	2.5P	15.5		Pathogenic	PS3, PS4 (moderate), PM2, PP3	Likely pathogenic (b)	2.5P	15.5		Pathogenic
p.His360Arg	1P	1.5P			2.5		Uncertain significance	PM2, PM3	Uncertain significance	1P	2.5		Uncertain significance
p.Asn367Ser	0.5P	1.5P			2		Uncertain significance	PM2, PM3, PP3	Uncertain significance	1P	2		Uncertain significance
p.Ile368Asn	0.5P	2P		1P	3.5		Uncertain significance	PS3, PM2, PP3	Likely pathogenic (b)	2.5P	5		Pathogenic
p.Leu369Pro	0.5P	1.5P		1P	3		Uncertain significance	PS3, PM2, PM3, PP3	Likely pathogenic (b)	2.5P	4		Likely Pathogenic
p.Gly386Ala	1P	2P		2.5P	5.5		Pathogenic	PS3, PM2, PP3	Likely pathogenic (b)	2.5P	5.5		Pathogenic
p.Cys388Arg	1P	3*2P	1P; 2 sibs homo &1P; 2 sibs homo	2.5P	11.5		Pathogenic	PS3, PM2, PS4 (moderate), PP3	Likely pathogenic (b)	2.5P	11.5		Pathogenic
p.Gly409Arg	0.5P	2P	1P; 2 sibs homo		4		Likely pathogenic	PM2, PS4 (moderate), PM5	Likely pathogenic (c)	2.5P	5.5		Pathogenic
p.Gly409Val	1P	2P		2.5P	5.5		Pathogenic	PS3, PM2, PP3	Likely pathogenic (b)	2.5P	5.5		Pathogenic
p.Pro416Leu	1P	2P			3		Uncertain significance	PM2, PM5	Uncertain significance	2.5P	5.5		Pathogenic
p.Pro416Arg	1P	2P+2P	1P; 2 sibs homo	1P	7		Pathogenic	PS3, PS4 (moderate), PM2, PM5, PP3	Pathogenic(d)	2.5P	7		Pathogenic
p.Glu417Gly	1P	2P		2.5P	5.5		Pathogenic	PS3, PM2, PP3	Likely pathogenic (b)	2.5P	5.5		Pathogenic
p.Ser419Pro	1P	2P	1P; 2 sibs homo		4		Likely pathogenic	PS4 (moderate), PM2, PP3	Uncertain significance	1P	5		Pathogenic
p.Trp437Arg	1P	2P			3		Uncertain significance	PM2, PP3	Uncertain significance	2.5P	5.5		Pathogenic
p.Arg464His	0.5P	1.5P			2		Uncertain significance	PM2, PM3	Uncertain significance	1P	3		Uncertain significance
p.Glu476Lys	2B+ 5B					7	Benign	BS1, BS2	Benign	2.5B		9.5	Benign
p.Leu489Pro	1P	1.5P		2.5P	5		Pathogenic	PS3, PM2, PM3, PP3	Likely pathogenic (b)	2.5P	5		Pathogenic
p.Arg501Gln	2B + 5B					7	Benign	BS1, BS2	Benign	2.5B		9.5	Benign
p.Asn521Thr	2B + 5B			1B		8	Benign	BA1, BS1, BS2. BS3	Benign	0		7	Benign
p.Ala537Thr	0.5P				0.5		Uncertain significance	PM2	Uncertain significance	2.5B	0.5	2.5	Uncertain significance

A = Sherloc points for evidence from population; B = Sherloc points for evidence from case reports; C = Sherloc points for evidence from segregation within family; D = Sherloc points for evidence from functional studies from literature (see Supplementary Table 2; SUM P = Cumulative pathogenic score; SUM B = Cumulative benign score; sibs = siblings; homo = homozygous; hetero = heterozygous.

Supplementary Table 2. Literature overview of biochemical effects of *PINK1* variants

PINK1 variant	Parkin recruitment	PINK1 kinase activity	Other	Score
p.Cys92Phe	Normal[1,2]	Normal PINK1 autophosphorylation[1]<br>Normal Parkin phosphorylation[3]	Normal dimeric PINK1-containing complex formation[4]<br>Increased ratio of full-length:cleaved PINK1[5]<br>Abnormally distributed and aggregated mitochondria[5]<br>Reduced CCCP-induced PINK1 OMM translocalization[6]	0
p.Gln115Leu	Normal[7]		Increased ratio of full-length:cleaved PINK1[5]<br>Abnormal PINK1 ubiquitination[8]	0
p.Cys125Gly	Impaired[2,7,9]	Decreased activity[10]	Normal PINK1 ubiquitination[8]<br>Diminished CCCP-induced full-length PINK1 accumulation[9]	2.5P
p.Gln126Pro	Impaired[2,7,9,11]		Abnormal PINK1 ubiquitination[8]<br>Diminished CCCP-induced full-length PINK1 accumulation[9]<br>Diminished CCCP-induced mitochondrial clearance[11]	2.5P
p.Thr145Met	Normal[7]		Normal PINK1 ubiquitination[8]	2.5B
p.Ala168Pro	Impaired[1,7,12]<br>Normal[2]	Impaired PINK1 autophosphorylation[1,13]<br>Impaired Parkin phosphorylation[3]	Impaired dimeric PINK1-containing complex formation[4]<br>Abnormal PINK1 ubiquitination[8]<br>Diminished CCCP-induced full-length PINK1 accumulation[14]	2.5P
p.Val170Gly	Impaired[15]		Diminished CCCP-induced full-length PINK1 accumulation[14]<br>Deficits in mitochondrial respiration[16,17]<br>Decreased mitochondrial membrane potential[17]<br>Impaired valinomycin-induced Parkin degradation[18]	2.5P
p.Pro196Leu	Impaired[2]		Abnormal PINK1 ubiquitination[8]	1P
p.Ala217Asp		Decreased activity[10]<br>Impaired PARIS phosphorylation[19]<br>Impaired Parkin phosphorylation[20]	Impaired mitochondrial clustering[21]	2.5P
p.Glu240Lys	Impaired[1]<br>Normal[22]	Decreased activity[10]<br>Impaired PINK1 autophosphorylation[1]<br>Impaired Parkin phosphorylation[3,20]<br>Impaired HDAC3 phosphorylation[23]	Impaired dimeric PINK1-containing complex formation[4]<br>Abrogated anti-apoptotic effects of PINK1[24,25]	2.5P
p.Ala244Gly			Abrogated anti-apoptotic effects of PINK1[26]	1P
p.His271Gln	Impaired[1,7,12]	Decreased activity[10]<br>Impaired PINK1 autophosphorylation[1]<br>Impaired Parkin phosphorylation[3]	Impaired dimeric PINK1-containing complex formation[4]<br>Abrogated anti-apoptotic effects of PINK1[25]<br>Impaired PINK1-Parkin interaction[27]	2.5P
p.Pro296Leu		Normal PINK1 autophosphorylation[28]	Impaired ubiquitin phosphorylation[28]	0
p.Gly309Asp	Impaired[1,2,7,12,29–31]<br>Normal[22]	Decreased activity[10]<br>Normal PINK1 autophosphorylation[1]<br>Impaired PINK1 autophosphorylation[13,28,32]<br>Impaired Parkin phosphorylation[3,33,34]<br>Impaired PARIS phosphorylation[19]<br>Impaired HDAC3 phosphorylation[23]<br>Impaired ubiquitin phosphorylation[28,33]<br>Impaired TRAP1 phosphorylation[35]<br>Impaired Drp1 phosphorylation[36]	Normal dimeric PINK1-containing complex formation[4]<br>Impaired mitochondrial clustering[21]<br>Abrogated anti-apoptotic effects of PINK1[25,37,38]<br>Impaired PINK1-Parkin interaction[27]<br>Altered TH expression[39,40]<br>Impaired CCCP-induced mitochondrial degradation[41]<br>Decreased mitochondrial membrane potential[42]<br>Perturbed mitochondrial dynamics[42]<br>Deficits in mitochondrial respiration[43]	2.5P
p.Thr313Met	Impaired[2,7,44]	Decreased activity[44]	Impaired CCCP-induced Parkin degradation[34]<br>Increasde cytotoxicity[45]	2.5P
p.Ala339Thr			Increased TH expression[39]<br>Abrogated anti-apoptotic effects of PINK1[46]	1P
p.Ala340Thr	Normal[7]			1B
p.Leu347Pro	Impaired[1,2,11,12,31]	Decreased activity[10]<br>Impaired PINK1 autophosphorylation[1,32,47]<br>Impaired Parkin phosphorylation[3,33]<br>Impaired PARIS phosphorylation[19]<br>Impaired HDAC3 phosphorylation[23]	Impaired dimeric PINK1-containing complex formation[4]<br>Diminished CCCP-induced mitochondrial clearance[11]<br>Impaired CCCP-induced mitochondrial clustering[21]<br>Abrogated anti-apoptotic effects of PINK1[25,38]<br>Impaired PINK1 protein stability[32,48]<br>Impaired CCCP-induced mitochondrial degradation[41]	2.5P

		Impaired TRAP1 phosphorylation[35]<br>Impaired ubiquitin phosphorylation[33]	Deficits in mitochondrial respiration[47]<br>Perturbed mitochondrial dynamics[49,50]<br>Impaired CCCP-induced Miro1 degradaton[51]	
p.Ile368Asn	Impaired[52]	Impaired ubiquitin phosphorylation[52]	Impaired PINK1 protein stability[52]	1P
p.Leu369Pro		Decreased activity[10]<br>Impaired Parkin phosphorylation[20]		1P
p.Gly386Ala	Impaired[1,2]	Decreased activity[10,53]<br>Impaired PINK1 autophosphorylation[1]<br>Impaired Parkin phosphorylation[3]		2.5P
p.Cys388Arg	Impaired[7]	Decreased activity[10]<br>Impaired Parkin phosphorylation[54]		2.5P
p.Gly409Val	Impaired[1,2,7]	Decreased activity[10,53]<br>Normal PINK1 autophosphorylation[1]<br>Impaired PINK1 autophosphorylation[28]<br>Impaired Parkin phosphorylation[3]<br>Impaired ubiquitin phosphorylation[28]	Normal dimeric PINK1-containing complex formation[4]<br>Impaired CCCP-induced mitochondrial degradation[41]	2.5P
p.Pro416Arg		Decreased activity[10]		1P
p.Glu417Gly	Impaired[1]	Decreased activity[10]<br>Impaired PINK1 autophosphorylation[1]<br>Impaired Parkin phosphorylation[3]	Impaired dimeric PINK1-containing complex formation[4]<br>Abrogated anti-apoptotic effects of PINK1[25]<br>Impaired PINK1-Parkin interaction[27]<br>Decreased mitochondrial membrane potential[42]<br>Perturbed mitochondrial dynamics[42]	2.5P
p.Leu489Pro		Decreased activity[10]<br>Impaired HDAC3 phosphorylation[23]	Abrogated anti-apoptotic effects of PINK1[24]	2.5P
p.Asn521Thr	Normal[2,7]			1B

Score = Pathogenic or Benign Sherloc score, based on the scheme shown in Supplementary Figure 2d.

References Supplementary Table 2

1. Okatsu K, Oka T, Iguchi M, et al. PINK1 autophosphorylation upon membrane potential dissipation is essential for Parkin recruitment to damaged mitochondria. *Nat Commun*. 2012;3(1):1-10. doi:10.1038/ncomms2016
2. Song S, Jang S, Park J, et al. Characterization of PINK1 (PTEN-induced putative kinase 1) mutations associated with parkinson disease in mammalian cells and drosophila. *J Biol Chem*. 2013;288(8):5660-5672. doi:10.1074/jbc.M112.430801
3. Iguchi M, Kujuro Y, Okatsu K, et al. Parkin-catalyzed ubiquitin-ester transfer is triggered by PINK1-dependent phosphorylation. *J Biol Chem*. 2013;288(30):22019-22032. doi:10.1074/jbc.M113.467530
4. Okatsu K, Uno M, Koyano F, et al. A Dimeric PINK1-containing Complex on Depolarized Mitochondria Stimulates Parkin Recruitment *. 2013. doi:10.1074/jbc.M113.509653
5. Deas E, Plun-Favreau H, Gandhi S, et al. PINK1 cleavage at position A103 by the mitochondrial protease PARL. *Hum Mol Genet*. 2011;20(5):867-879. doi:10.1093/hmg/ddq526
6. Fallaize D, Chin LS, Li L. Differential submitochondrial localization of PINK1 as a molecular switch for mediating distinct mitochondrial signaling pathways. *Cell Signal*. 2015;27(12):2543-2554. doi:10.1016/j.cellsig.2015.09.020
7. Narendra DP, Wang C, Youle RJ, Walker JE. PINK1 rendered temperature sensitive by disease-associated and engineered mutations. *Hum Mol Genet*. 2013;22(13):2572-2589. doi:10.1093/hmg/ddt106
8. Guardia-Laguarta C, Liu Y, Lauritzen KH, et al. PINK1 Content in Mitochondria is Regulated by ER-Associated Degradation. *J Neurosci*. 2019;39(36):7074-7085. doi:10.1523/JNEUROSCI.1691-18.2019
9. Sekine S, Wang C, Sideris DP, Bunker E, Zhang Z, Youle RJ. Reciprocal Roles of Tom7 and OMA1 during Mitochondrial Import and Activation of PINK1. *Mol Cell*. 2019;73(5):1028-1043.e5. doi:10.1016/j.molcel.2019.01.002
10. Woodroof HI, Pogson JH, Begley M, et al. Discovery of catalytically active orthologues of the Parkinson's disease kinase PINK1: analysis of substrate specificity and impact of mutations. *Open Biol*. 2011;1(3):110012. doi:10.1098/rsob.110012
11. Geisler S, Holmström KM, Treis A, et al. The PINK1/Parkin-mediated mitophagy is compromised by PD-associated mutations. *Autophagy*. 2010;6(7):871-878. doi:10.4161/auto.6.7.13286
12. Narendra DP, Jin SM, Tanaka A, et al. PINK1 is selectively stabilized on impaired mitochondria to activate Parkin. *PLoS Biol*. 2010;8(1). doi:10.1371/journal.pbio.1000298
13. Silvestri L, Caputo V, Bellacchio E, et al. Mitochondrial import and enzymatic activity of PINK1 mutants associated to recessive parkinsonism. *Hum Mol Genet*. 2005;14(22):3477-3492. doi:10.1093/hmg/ddi377
14. Gao F, Zhang Y, Hou X, Tao Z, Ren H, Wang G. Dependence of PINK1 accumulation on mitochondrial redox system. *Aging Cell*. 2020;19(9). doi:10.1111/acel.13211
15. Seibler P, Graziotto J, Jeong H, Simunovic F, Klein C, Krainc D. Mitochondrial parkin recruitment is impaired in neurons derived from mutant PINK1 induced pluripotent stem cells. *J Neurosci*. 2011;31(16):5970-5976. doi:10.1523/JNEUROSCI.4441-10.2011
16. Grünewald A, Gegg ME, Taanman JW, et al. Differential effects of PINK1 nonsense and missense mutations on mitochondrial function and morphology. *Exp Neurol*. 2009;219(1):266-273. doi:10.1016/j.expneurol.2009.05.027
17. Morais VA, Haddad D, Craessaerts K, et al. PINK1 loss-of-function mutations affect mitochondrial complex I activity via NdufA10 ubiquinone uncoupling. *Science (80-)*. 2014;344(6180):203-207. doi:10.1126/science.1249161
18. Rakovic A, Grünewald A, Seibler P, et al. Effect of endogenous mutant and wild-type PINK1 on Parkin in fibroblasts from Parkinson disease patients. *Hum Mol Genet*. 2010;19(16):3124-3137. doi:10.1093/hmg/ddq215
19. Lee Y, Stevens DA, Kang SU, et al. PINK1 Primes Parkin-Mediated Ubiquitination of PARIS in Dopaminergic Neuronal Survival. *Cell Rep*. 2017;18(4):918-932. doi:10.1016/j.celrep.2016.12.090
20. Kumar A, Tamjar J, Waddell AD, et al. Structure of PINK1 and mechanisms of Parkinson's disease-associated mutations. *Elife*. 2017;6. doi:10.7554/eLife.29985
21. Vives-Bauza C, Zhou C, Huang Y, et al. PINK1-dependent recruitment of Parkin to mitochondria in mitophagy. *Proc Natl Acad Sci U S A*. 2010;107(1):378-383. doi:10.1073/pnas.0911187107
22. Matsuda N, Sato S, Shiba K, et al. PINK1 stabilized by mitochondrial depolarization recruits Parkin to damaged mitochondria and activates latent Parkin for mitophagy. *J Cell Biol*. 2010;189(2):211-221. doi:10.1083/jcb.200910140
23. Choi HK, Choi Y, Kang HB, et al. Pink1 positively regulates HDAC3 to suppress dopaminergic neuronal cell death. *Hum Mol Genet*. 2015;24(4):1127-1141. doi:10.1093/hmg/ddu526
24. Petit A, Kawarai T, Paitel E, et al. Wild-type PINK1 prevents basal and induced neuronal apoptosis, a protective effect abrogated by Parkinson disease-related mutations. *J Biol Chem*. 2005;280(40):34025-34032. doi:10.1074/jbc.M505143200
25. Wang HL, Chou AH, Yeh TH, et al. PINK1 mutants associated with recessive Parkinson's disease are defective in inhibiting mitochondrial release of cytochrome c. *Neurobiol Dis*. 2007;28(2):216-226. doi:10.1016/j.nbd.2007.07.010

26. Gelmetti V, Ferraris A, Brusa L, et al. Late onset sporadic Parkinson's disease caused by *PINK1* mutations: Clinical and functional study. *Mov Disord*. 2008;23(6):881-885. doi:10.1002/mds.21960
27. Shiba K, Arai T, Sato S, et al. Parkin stabilizes PINK1 through direct interaction. *Biochem Biophys Res Commun*. 2009;383(3):331-335. doi:10.1016/j.bbrc.2009.04.006
28. Schubert AF, Gladkova C, Pardon E, et al. Structure of PINK1 in complex with its substrate ubiquitin. *Nature*. 2017;552(7683):1-28. doi:10.1038/nature24645
29. Hertz NT, Berthet A, Sos ML, et al. A neo-substrate that amplifies catalytic activity of parkinson's-disease- related kinase PINK1. *Cell*. 2013;154(4):737-747. doi:10.1016/j.cell.2013.07.030
30. Murata H, Sakaguchi M, Kataoka K, Huh NH. SARM1 and TRAF6 bind to and stabilize PINK1 on depolarized mitochondria. *Mol Biol Cell*. 2013;24(18):2772-2784. doi:10.1091/mbc.E13-01-0016
31. Um JW, Stichel-Gunkel C, Lübbert H, Lee G, Chung KC. Molecular interaction between parkin and PINK1 in mammalian neuronal cells. *Mol Cell Neurosci*. 2009;40(4):421-432. doi:10.1016/j.mcn.2008.12.010
32. Beilina A, Van Der Brug M, Ahmad R, et al. Mutations in PTEN-induced putative kinase 1 associated with recessive parkinsonism have differential effects on protein stability. *Proc Natl Acad Sci U S A*. 2005;102(16):5703-5708. doi:10.1073/pnas.0500617102
33. Sha D, Chin LS, Li L. Phosphorylation of parkin by Parkinson disease-linked kinase PINK1 activates parkin E3 ligase function and NF-κB signaling. *Hum Mol Genet*. 2009;19(2):352-363. doi:10.1093/hmg/ddp501
34. Xiong H, Wang D, Chen L, et al. Parkin, PINK1, and DJ-1 form a ubiquitin E3 ligase complex promoting unfolded protein degradation. *J Clin Invest*. 2009;119(3):650-660. doi:10.1172/JCI37617
35. Pridgeon JW, Olzmann JA, Chin L-S, Li L. PINK1 Protects against Oxidative Stress by Phosphorylating Mitochondrial Chaperone TRAP1. Zoghbi HY, ed. *PLoS Biol*. 2007;5(7):e172. doi:10.1371/journal.pbio.0050172
36. Han H, Tan J, Wang R, et al. PINK 1 phosphorylates Drp1 S616 to regulate mitophagy-independent mitochondrial dynamics . *EMBO Rep*. 2020;21(8). doi:10.15252/embr.201948686
37. Murata H, Sakaguchi M, Jin Y, et al. A new cytosolic pathway from a Parkinson disease-associated kinase, BRPK/PINK1: Activation of AKT via MTORC2. *J Biol Chem*. 2011;286(9):7182-7189. doi:10.1074/jbc.M110.179390
38. Sánchez-Mora RM, Arboleda H, Arboleda G. PINK1 overexpression protects against C2-ceramide-induced CAD cell death through the PI3K/AKT pathway. *J Mol Neurosci*. 2012;47(3):582-594. doi:10.1007/s12031-011-9687-z
39. Zhou ZD, Refai FS, Xie SP, et al. Mutant PINK1 upregulates tyrosine hydroxylase and dopamine levels, leading to vulnerability of dopaminergic neurons. *Free Radic Biol Med*. 2014;68:220-233. doi:10.1016/j.freeradbiomed.2013.12.015
40. Lu L, Jia H, Gao G, et al. Pink1 Regulates Tyrosine Hydroxylase Expression and Dopamine Synthesis. *J Alzheimer's Dis*. 2018;63(4):1361-1371. doi:10.3233/JAD-170832
41. Kawajiri S, Saiki S, Sato S, et al. PINK1 is recruited to mitochondria with parkin and associates with LC3 in mitophagy. *FEBS Lett*. 2010;584(6):1073-1079. doi:10.1016/j.febslet.2010.02.016
42. Wang HL, Chou AH, Wu AS, et al. PARK6 PINK1 mutants are defective in maintaining mitochondrial membrane potential and inhibiting ROS formation of substantia nigra dopaminergic neurons. *Biochim Biophys Acta - Mol Basis Dis*. 2011;1812(6):674-684. doi:10.1016/j.bbadis.2011.03.007
43. Morais VA, Verstreken P, Roethig A, et al. Parkinson's disease mutations in PINK1 result in decreased Complex I activity and deficient synaptic function. *EMBO Mol Med*. 2009;1(2):99-111. doi:10.1002/emmm.200900006
44. Guo J feng, Yao L yan, Sun Q ying, et al. Identification of Ser465 as a novel PINK1 autophosphorylation site. *Transl Neurodegener*. 2017;6(1):34. doi:10.1186/s40035-017-0103-7
45. Matenia D, Hempp C, Timm T, Eikhof A, Mandelkow EM. Microtubule Affinity-regulating Kinase 2 (MARK2) turns on phosphatase and tensin homolog (PTEN)-induced kinase 1 (PINK1) at Thr-313, a mutation site in Parkinson disease: Effects on mitochondrial transport. *J Biol Chem*. 2012;287(11):8174-8186. doi:10.1074/jbc.M111.262287
46. Tan EK, Refai FS, Siddique M, et al. Clinically reported heterozygous mutations in the PINK1 kinase domain exert a gene dosage effect. *Hum Mutat*. 2009;30(11):1551-1557. doi:10.1002/humu.21108
47. Liu W, Vives-Bauza C, Acín-Peréz- R, et al. PINK1 Defect Causes Mitochondrial Dysfunction, Proteasomal Deficit and α-Synuclein Aggregation in Cell Culture Models of Parkinson's Disease. Cookson MR, ed. *PLoS One*. 2009;4(2):e4597. doi:10.1371/journal.pone.0004597
48. Moriwaki Y, Kim YJ, Ido Y, et al. L347P PINK1 mutant that fails to bind to Hsp90/Cdc37 chaperones is rapidly degraded in a proteasome-dependent manner. *Neurosci Res*. 2008;61(1):43-48. doi:10.1016/j.neures.2008.01.006
49. Yu W, Sun Y, Guo S, Lu B. The PINK1/Parkin pathway regulates mitochondrial dynamics and function in mammalian hippocampal and dopaminergic neurons. *Hum Mol Genet*. 2011;20(16):3227-3240. doi:10.1093/hmg/ddr235
50. Cui M, Tang X, Christian W V., Yoon Y, Tieu K. Perturbations in mitochondrial dynamics induced by human mutant PINK1 can be rescued by the mitochondrial division inhibitor mdivi-1. *J Biol Chem*. 2010;285(15):11740-11752. doi:10.1074/jbc.M109.066662
51. Liu S, Sawada T, Lee S, et al. Parkinson's Disease–Associated Kinase PINK1 Regulates Miro Protein Level and Axonal Transport of Mitochondria. Copenhaver GP, ed. *PLoS Genet*. 2012;8(3):e1002537. doi:10.1371/journal.pgen.1002537
52. Ando M, Fiesel FC, Hudec R, et al. The PINK1 p.I368N mutation affects protein stability and ubiquitin kinase activity. *Mol Neurodegener*. 2017;12(1):32. doi:10.1186/s13024-017-0174-z
53. Sim CH, Lio DSS, Mok SS, et al. C-terminal truncation and Parkinson's disease-associated mutations down-regulate the protein serine/threonine kinase activity of PTEN-induced kinase-1. *Hum Mol Genet*. 2006;15(21):3251-3262. doi:10.1093/hmg/ddl398
54. Shiba-Fukushima K, Imai Y, Yoshida S, et al. PINK1-mediated phosphorylation of the Parkin ubiquitin-like domain primes mitochondrial translocation of Parkin and regulates mitophagy. *Sci Rep*. 2012;2(1):1-8. doi:10.1038/srep01002

Supplementary Table 3. Mutagenesis primer list for the generation of *PINK1* variants

Primer name	Variant	Direction	Sequence (5'>3')
Mut_PINK1_L11M_For	L11M	Forward	CTGGGCCGCGGCATGCAGCTGGGTC
Mut_PINK1_L11M_Rev	L11M	Reverse	GACCCAGCTGCATGCCGCGGCCCAG
Mut_PINK1_G30R_For	G30R	Forward	GGCCGGGCCTACCGCTTGGGGCGGC
Mut_PINK1_G30R_Rev	G30R	Reverse	GCCGCCCCAAGCGGTAGGCCCGGCC
Mut_PINK1_L77P_For	L77P	Forward	CGGTGGCCGGGCCGGCGGCGCGGTTG
Mut_PINK1_L77P_Rev	L77P	Reverse	CAACCGCGCCGCCGGCCCGGCCACCG
Mut_PINK1_C92F_For	C92F	Forward	GGGCCTGGGGCTTCGCGGGCCCTTG
Mut_PINK1_C92F_Rev	C92F	Reverse	CAAGGGCCCGCGAAGCCCCAGGCCC
Mut_PINK1_Q115L_For	Q115L	Forward	CATCGAGGAAAAACTGGCGGAGAGCCGG
Mut_PINK1_Q115L_Rev	Q115L	Reverse	CCGGCTCTCCGCCAGTTTTTCCTCGATG
Mut_PINK1_C125G_For	C125G	Forward	GCGGTCTCGGCCGGTCAGGAGATCC
Mut_PINK1_C125G_Rev	C125G	Reverse	GGATCTCCTGACCGGCCGAGACCGC
Mut_PINK1_Q126P_For	Q126P	Forward	GTCTCGGCCTGTCCGGAGATCCAGGC
Mut_PINK1_Q126P_Rev	Q126P	Reverse	GCCTGGATCTCCGGACAGGCCGAGAC
Mut_PINK1_A168P_For	A168P	Forward	GTAAGGGCTGCAGTCCTGCTGTGTATGAAG
Mut_PINK1_A168P_Rev	A168P	Reverse	CTTCATACACAGCAGGACTGCAGCCCTTAC
Mut_PINK1_V170G_For	V170G	Forward	GCAGTGCTGCTGGGTATGAAGCCAC
Mut_PINK1_V170G_Rev	V170G	Reverse	GTGGCTTCATACCCAGCAGCACTGC
Mut_PINK1_A217D_For	A217D	Forward	CCTTCCCCTTGGACATCAAGATGATG
Mut_PINK1_A217D_Rev	A217D	Reverse	CATCATCTTGATGTCCAAGGGGAAGG
Mut_PINK1_E240K_For	E240K	Forward	CACAATGAGCCAGAAGCTGGTCCCAG
Mut_PINK1_E240K_Rev	E240K	Reverse	CTGGGACCAGCTTCTGGCTCATTGTG
Mut_PINK1_A244G_For	A244G	Forward	GAGCTGGTCCCAGGGAGCCGAGTGGC
Mut_PINK1_A244G_Rev	A244G	Reverse	GCCACTCGGCTCCCTGGGACCAGCTC
Mut_PINK1_L249V_For	L249V	Forward	CGAGCCGAGTGGCCGTGGCTGGGGAGTATG
Mut_PINK1_L249V_Rev	L249V	Reverse	CATACTCCCCAGCCACGGCCACTCGGCTCG
Mut_PINK1_H271Q_For	H271Q	Forward	CAACTAGCCCCTCAACCCAACATCATCC
Mut_PINK1_H271Q_Rev	H271Q	Reverse	GGATGATGTTGGGTTGAGGGGCTAGTTG
Mut_PINK1_G309D_For	G309D	Forward	CTGAAGGCCTGGACCATGGCCGGAC
Mut_PINK1_G309D_Rev	G309D	Reverse	GTCCGGCCATGGTCCAGGCCTTCAG
Mut_PINK1_T313M_For	T313M	Forward	CTGGGCCATGGCCGGATGCTGTTCCTCGTTATG
Mut_PINK1_T313M_Rev	T313M	Reverse	CATAACGAGGAACAGCATCCGGCCATGGCCCAG
Mut_PINK1_A339T_For	A339T	Forward	CAGCCCCCGCCTCACCGCCATGATGC
Mut_PINK1_A339T_Rev	A339T	Reverse	GCATCATGGCGGTGAGGCGGGGGCTG
Mut_PINK1_A340T_For	A340T	Forward	CCCCGCCTCGCCACCATGATGCTGC
Mut_PINK1_A340T_Rev	A340T	Reverse	GCAGCATCATGGTGGCGAGGCGGGG
Mut_PINK1_L347P_For	L347P	Forward	CTGCTGCAGCTGCCGGAAGGCGTGGAC
Mut_PINK1_L347P_Rev	L347P	Reverse	GTCCACGCCTTCCGGCAGCTGCAGCAG
Mut_PINK1_G386A_For	G386A	Forward	GATCGCAGATTTTGCCTGCTGCCTGGCTG
Mut_PINK1_G386A_Rev	G386A	Reverse	CAGCCAGGCAGCAGGCAAAATCTGCGATC
Mut_PINK1_C388R_For	C388R	Forward	GATTTTGGCTGCCGCCTGGCTGATG
Mut_PINK1_C388R_Rev	C388R	Reverse	CATCAGCCAGGCGGCAGCCAAAATC
Mut_PINK1_P416R_For	P416R	Forward	GTCTGATGGCCCGAGAGGTGTCCAC
Mut_PINK1_P416R_Rev	P416R	Reverse	GTGGACACCTCTCGGGCCATCAGAC
Mut_PINK1_S419P_For	S419P	Forward	GCCCCAGAGGTGCCCACGGCCCGTC
Mut_PINK1_S419P_Rev	S419P	Reverse	GACGGGCCGTGGGCACCTCTGGGGC
Mut_PINK1_E476K_For	E476K	Forward	CCTGCACTGCCCAAGTCAGTGCCTC
Mut_PINK1_E476K_Rev	E476K	Reverse	GAGGCACTGACTTGGGCAGTGCAGG
Mut_PINK1_R501Q_For	R501Q	Forward	GAGACCATCTGCCCAAGTAGCCGCAAATG
Mut_PINK1_R501Q_Rev	R501Q	Reverse	CATTTGCGGCTACTTGGGCAGATGGTCTC
Mut_PINK1_N521T_For	N521T	Forward	CTAGCCCTGAAGACTCTGAAGTTAGAC
Mut_PINK1_N521T_Rev	N521T	Reverse	GTCTAACTTCAGAGTCTTCAGGGCTAG
Mut_PINK1_I131V_For	I131V	Forward	CAGGAGATCCAGGCAGTTTTTACCCAGAAAAG
Mut_PINK1_I131V_Rev	I131V	Reverse	CTTTTCTGGGTAAAAACTGCCTGGATCTCCTG
Mut_PINK1_C323Y_For	C323Y	Forward	GAAGAACTATCCCTATACCCTGCGCCAG
Mut_PINK1_C323Y_Rev	C323Y	Reverse	CTGGCGCAGGGTATAGGGATAGTTCTTC
Mut_PINK1_A537T_For	A537T	Forward	CCAACAATCGGCCACCACTTTGTTGGC
Mut_PINK1_A537T_Rev	A537T	Reverse	GCCAACAAAGTGGTGGCCGATTGTTGG
Mut_PINK1_G189R_For	G189R	Forward	GTGACAAAGAGCACCAGGTTGCTTCCAGGG
Mut_PINK1_G189R_Rev	G189R	Reverse	CCCTGGAAGCAACCTGGTGCTCTTTGTCAC
Mut_PINK1_P196L_For	P196L	Forward	CAGGGAGAGGCCTAGGTACCAGTGC
Mut_PINK1_P196L_Rev	P196L	Reverse	GCACTGGTACCTAGGCCTCTCCCTG
Mut_PINK1_T257I_For	T257I	Forward	GGAGTATGGAGCAGTCATTTACAGAAAATCCAAG
Mut_PINK1_T257I_Rev	T257I	Reverse	CTTGGATTTTCTGTAAATGACTGCTCCATACTCC
Mut_PINK1_P296L_For	P296L	Forward	CTGGTCGACTACCTTGATGTGCTGCC
Mut_PINK1_P296L_Rev	P296L	Reverse	GGCAGCACATCAAGGTAGTCGACCAG
Mut_PINK1_H360R_For	H360R	Forward	CAACAGGGCATCGCGCGCAGAGACCTGAAATC
Mut_PINK1_H360R_Rev	H360R	Reverse	GATTTCAGGTCTCTGCGCGCGATGCCCTGTTG
Mut_PINK1_I368N_For	I368N	Forward	GAAATCCGACAACAACCTTGTGGAGCTG
Mut_PINK1_I368N_Rev	I368N	Reverse	CAGCTCCACAAGGTTGTTGTCGGATTTC
Mut_PINK1_L369P_For	L369P	Forward	CCGACAACATCCCTGTGGAGCTGGAC
Mut_PINK1_L369P_Rev	L369P	Reverse	GTCCAGCTCCACAGGGATGTTGTCGG
Mut_PINK1_G409V_For	G409V	Forward	GTGGATCGGGGCGTAAACGGCTGTCTG
Mut_PINK1_G409V_Rev	G409V	Reverse	CAGACAGCCGTTTACGCCCCGATCCAC
Mut_PINK1_W437R_For	W437R	Forward	CAAGGCTGATGCCCGGGCAGTGGGAG
Mut_PINK1_W437R_Rev	W437R	Reverse	CTCCCACTGCCCGGGCATCAGCCTTG
Mut_PINK1_T145M_For	T145M	Forward	GACCCGTTGGACATGAGACGCTTGCAG
Mut_PINK1_T145M_Rev	T145M	Reverse	CTGCAAGCGTCTCATGTCCAACGGGTC
Mut_PINK1_M237V_For	M237V	Forward	CCATCTTGAACACAGTGAGCCAGGAGCTG
Mut_PINK1_M237V_Rev	M237V	Reverse	CAGCTCCTGGCTCACTGTGTTCAAGATGG
Mut_PINK1_S73L_For	S73L	Forward	CTTCTTCCGCCAGTTGGTGGCCGGGCTG
Mut_PINK1_S73L_Rev	S73L	Reverse	CAGCCCGGCCACCAACTGGCGGAAGAAG

Mut_PINK1_R152W_For	R152W	Forward	GCTTGCAGGGCTTTTGGCTGGAGGAGTATC
Mut_PINK1_R152W_Rev	R152W	Reverse	GATACTCCTCCAGCCAAAAGCCCTGCAAGC
Mut_PINK1_R279H_For	R279H	Forward	CATCCGGGTTCTCCACGCCTTCACCTCTTC
Mut_PINK1_R279H_Rev	R279H	Reverse	GAAGAGGTGAAGGCGTGGAGAACCCGGATG
Mut_PINK1_V317I_For	V317I	Forward	CGGACGCTGTTCCTCATTATGAAGAACTATC
Mut_PINK1_V317I_Rev	V317I	Reverse	GATAGTTCTTCATAATGAGGAACAGCGTCCG
Mut_PINK1_N367S_For	N367S	Forward	CCTGAAATCCGACAGCATCCTTGTGGAG
Mut_PINK1_N367S_Rev	N367S	Reverse	CTCCACAAGGATGCTGTCGGATTTCAGG
Mut_PINK1_G409R_For	G409R	Forward	CGTGGATCGGGGCAGAAACGGCTGTCTG
Mut_PINK1_G409R_Rev	G409R	Reverse	CAGACAGCCGTTTCTGCCCCGATCCACG
Mut_PINK1_P416L_For	P416L	Forward	CTGTCTGATGGCCCTAGAGGTGTCCACG
Mut_PINK1_P416L_Rev	P416L	Reverse	CGTGGACACCTCTAGGGCCATCAGACAG
Mut_PINK1_E417G_For	E417G	Forward	CTGATGGCCCCAGGGGTGTCCACGGCCC
Mut_PINK1_E417G_Rev	E417G	Reverse	GGGCCGTGGACACCCCTGGGGCCATCAG
Mut_PINK1_R464H_For	R464H	Forward	CCACCTTGAAAGCCACAGCTACCAAGAGG
Mut_PINK1_R464H_Rev	R464H	Reverse	CCTCTTGGTAGCTGTGGCTTTCAAGGTGG
Mut_PINK1_L489P_For	L489P	Forward	GTTGGTGAGGGCACCGCTCCAGCGAGAGGC
Mut_PINK1_L489P_Rev	L489P	Reverse	GCCTCTCGCTGGAGCGGTGCCCTCACCAAC
