# Supplementary material for: Systematic analysis of PINK1 variants of unknown significance shows intact mitophagy function for most variants
Source: NPJ Parkinsons Dis. 2021 Dec 10;7:113. doi: 10.1038/s41531-021-00258-8 (PMC8664852; doi:10.1038/s41531-021-00258-8)
Supplement: Supplementary file 2 — Reporting Summary [file 41531_2021_258_MOESM2_ESM.pdf]

nature portfolio

Corresponding author(s): Dineke Verbeek

Last updated by author(s): Sep 10, 2021

Reporting Summary

Nature Portfolio wishes to improve the reproducibility of the work that we publish. This form provides structure for consistency and transparency in reporting. For further information on Nature Portfolio policies, see our Editorial Policies and the Editorial Policy Checklist.

Statistics

For all statistical analyses, confirm that the following items are present in the figure legend, table legend, main text, or Methods section.

n/a	Confirmed	
☐	☒	The exact sample size (*n*) for each experimental group/condition, given as a discrete number and unit of measurement
☐	☒	A statement on whether measurements were taken from distinct samples or whether the same sample was measured repeatedly
☐	☒	The statistical test(s) used AND whether they are one- or two-sided<br>*Only common tests should be described solely by name; describe more complex techniques in the Methods section.*
☒	☐	A description of all covariates tested
☒	☐	A description of any assumptions or corrections, such as tests of normality and adjustment for multiple comparisons
☒	☐	A full description of the statistical parameters including central tendency (e.g. means) or other basic estimates (e.g. regression coefficient) AND variation (e.g. standard deviation) or associated estimates of uncertainty (e.g. confidence intervals)
☒	☐	For null hypothesis testing, the test statistic (e.g. *F*, *t*, *r*) with confidence intervals, effect sizes, degrees of freedom and *P* value noted<br>*Give P values as exact values whenever suitable.*
☒	☐	For Bayesian analysis, information on the choice of priors and Markov chain Monte Carlo settings
☒	☐	For hierarchical and complex designs, identification of the appropriate level for tests and full reporting of outcomes
☒	☐	Estimates of effect sizes (e.g. Cohen's *d*, Pearson's *r*), indicating how they were calculated

Our web collection on statistics for biologists contains articles on many of the points above.

Software and code

Policy information about availability of computer code

Data collection	MDSgene and Clinvar (websites, access date: 15 sept 2020)<br>gnomAD version 2.1.1
Data analysis	Kaluza Analysis Software<br>ImageJ (version 2.0.0-rc-69/1.52p)<br>R (version 1.3.959)

For manuscripts utilizing custom algorithms or software that are central to the research but not yet described in published literature, software must be made available to editors and reviewers. We strongly encourage code deposition in a community repository (e.g. GitHub). See the Nature Portfolio guidelines for submitting code & software for further information.

Data

Policy information about availability of data

All manuscripts must include a data availability statement. This statement should provide the following information, where applicable:

- Accession codes, unique identifiers, or web links for publicly available datasets
- A description of any restrictions on data availability
- For clinical datasets or third party data, please ensure that the statement adheres to our policy

The datasets generated during and/or analysed during the current study are available from the corresponding author on reasonable request.

Field-specific reporting

Please select the one below that is the best fit for your research. If you are not sure, read the appropriate sections before making your selection.

☒ Life sciences ☐ Behavioural & social sciences ☐ Ecological, evolutionary & environmental sciences

For a reference copy of the document with all sections, see nature.com/documents/nr-reporting-summary-flat.pdf

Life sciences study design

All studies must disclose on these points even when the disclosure is negative.

Sample size	No sample-size calculations were made. Each data point shown in this study is a separate biological replicate. As per usual in cell biological studies, at least three independent replicates were made.
Data exclusions	No data was excluded
Replication	Independent replicates were experimentally sound and provided results that were similar as expected
Randomization	In each experiment, the variants were ordered based on their aminoacid number. Experiments were performed over multiple days and analysed approximately in sets of 12.
Blinding	Immunofluorescence images were analysed with a blinded observed. Images-files were renamed such that the observer could not determine the origin of the sample.

Reporting for specific materials, systems and methods

We require information from authors about some types of materials, experimental systems and methods used in many studies. Here, indicate whether each material, system or method listed is relevant to your study. If you are not sure if a list item applies to your research, read the appropriate section before selecting a response.

Materials & experimental systems

n/a	Involved in the study	
☐	☒	Antibodies
☐	☒	Eukaryotic cell lines
☒	☐	Palaeontology and archaeology
☒	☐	Animals and other organisms
☒	☐	Human research participants
☒	☐	Clinical data
☒	☐	Dual use research of concern

Methods

n/a	Involved in the study	
☒	☐	ChIP-seq
☐	☒	Flow cytometry
☒	☐	MRI-based neuroimaging

Antibodies

Antibodies used	rat anti-HA IgG monoclonal antibody (Immunofluorescence, Roche, 11867423001, 3F10)<br>goat anti-Rat IgG (H+L) Alexa Fluor 594 secondary antibody (Immunofluorescence, Invitrogen, A-11007)<br>mouse anti-beta-actin (Western blotting, MP Biomedicals 8691001, Clone C4)<br>mouse anti-Parkin (Western blotting, Santa-cruz Biotechnology, sc-32282, PRK8)<br>rabbit anti-PINK1 (Western blotting, Cell signaling, #6946, D8G3)<br>HRP-conjugated goat anti-mouse IgG (H+L) (Western blotting, Bio-Rad,#1706516)<br>HRP-conjugated goat anti-rabbit IgG (H+L) (Western blotting, Bio-Rad, #1706515)
Validation	Each antibody has been cited numerous times at CiteAb and showed at the correct size

Eukaryotic cell lines

Policy information about cell lines

Cell line source(s)	ATCC
Authentication	None of the cell lines were authenticated
Mycoplasma contamination	All cell lines were regularly tested negative for mycoplasma contamination

Commonly misidentified lines
(See ICLAC register)

Name any commonly misidentified cell lines used in the study and provide a rationale for their use.

Flow Cytometry

Plots

Confirm that:

☒ The axis labels state the marker and fluorochrome used (e.g. CD4-FITC).

☒ The axis scales are clearly visible. Include numbers along axes only for bottom left plot of group (a 'group' is an analysis of identical markers).

☒ All plots are contour plots with outliers or pseudocolor plots.

☒ A numerical value for number of cells or percentage (with statistics) is provided.

Methodology

Sample preparation	Cells were dissociated with trypsin-EDTA, washed and resuspended in DAPI containing medium. Cells were then measured.
Instrument	Novocyte Quanteon flow cytometer (Agilent)
Software	Kaluza Analysis Software
Cell population abundance	Cells were stably expressing the mt-mKeima construct. Nearly all cells showed fluorescence emission at the expected wavelengths.
Gating strategy	A first gate was used to only select single cells. A second was used to determine DAPI negative cells (i.e. living cells). A third gate was used to determine the population cells with no mitophagy induction (DMSO ctrl). This last gate was used to determine mitophagy induction

☐ Tick this box to confirm that a figure exemplifying the gating strategy is provided in the Supplementary Information.
